# Biodegradable and biocompatible exceedingly small magnetic iron oxide nanoparticles for *T*_1_-weighted magnetic resonance imaging of tumors

**DOI:** 10.1186/s12951-022-01562-y

**Published:** 2022-07-30

**Authors:** Xuanyi Lu, Huimin Zhou, Zhiyu Liang, Jie Feng, Yudie Lu, Lin Huang, Xiaozhong Qiu, Yikai Xu, Zheyu Shen

**Affiliations:** 1 Biomaterials Research Center, School of Biomedical Engineering, Southern Medical University, 1023 Shatai South Road, Guangzhou, 510515 Guangdong China; 2 Guangdong Provincial Key Laboratory of Construction and Detection in Tissue Engineering, School of Basic Medical Sciences, Southern Medical University, 1023 Shatai South Road, Guangzhou, 510515 Guangdong China; 3 Medical Imaging Center, Nanfang Hospital, School of Biomedical Engineering, Southern Medical University, 1023 Shatai South Road, Guangzhou, 510515 Guangdong China

**Keywords:** Magnetic resonance imaging (MRI), Contrast agents (CAs), Exceedingly small magnetic iron oxide nanoparticles (ES-MIONs), Poly (aspartic acid) (PASP), Biodegradable

## Abstract

Magnetic resonance imaging (MRI) has been widely using in clinical diagnosis, and contrast agents (CAs) can improve the sensitivity MRI. To overcome the problems of commercial Gd chelates-based *T*_1_ CAs, commercial magnetic iron oxide nanoparticles (MIONs)-based *T*_2_ CAs, and reported exceedingly small MIONs (ES-MIONs)-based *T*_1_ CAs, in this study, a facile co-precipitation method was developed to synthesize biodegradable and biocompatible ES-MIONs with excellent water-dispersibility using poly (aspartic acid) (PASP) as a stabilizer for *T*_1_-weighted MRI of tumors. After optimization of the synthesis conditions, the final obtained ES-MION9 with 3.7 nm of diameter has a high *r*_1_ value (7.0 ± 0.4 mM^−1^ s^−1^) and a low *r*_2_/*r*_1_ ratio (4.9 ± 0.6) at 3.0 T. The ES-MION9 has excellent water dispersibility because of the excessive –COOH from the stabilizer PASP. The pharmacokinetics and biodistribution of ES-MION9 in vivo demonstrate the better tumor targetability and MRI time window of ES-MION9 than commercial Gd chelates. *T*_1_-weighted MR images of aqueous solutions, cells and tumor-bearing mice at 3.0 T or 7.0 T demonstrate that our ES-MION9 has a stronger capability of enhancing the MRI contrast comparing with the commercial Gd chelates. The MTT assay, live/dead staining of cells, and H&E-staining indicate the non-toxicity and biosafety of our ES-MION9. Consequently, the biodegradable and biocompatible ES-MION9 with excellent water-dispersibility is an ideal *T*_1_-weighted CAs with promising translational possibility to compete with the commercial Gd chelates.

**Supplementary Information:**

The online version contains supplementary material available at 10.1186/s12951-022-01562-y.

## Introduction

Magnetic resonance imaging (MRI) has been widely using in clinical diagnosis and prognosis observation to distinguish lesions from normal tissues, especially for the diagnosis of tumors, because of its obvious superiorities, including high soft tissue contrast, high spatial resolution, non-invasion and non-radiation [1–4]. Contrast agents (CAs) play an indispensable role to enhance the sensitivity of MRI. *T*_1_-weighted CAs (i.e., positive CAs) can shorten the proton’s longitudinal relaxation time (*T*_1_) to produce brighter images, while *T*_2_-weighted CAs (i.e., negative CAs) can shorten proton’s transverse relaxation time (*T*_2_) to generate darker images [5–8]. Currently, most clinical *T*_1_ CAs are gadolinium (Gd) chelates, including Magnevist (Gd-DTPA), Gadavist (Gd-DO3A-Butriol), Dotarem (Gd-DOTA), Eovist (Gd-EOB-DTPA), Omniscan (Gd-DTPA-BMA), and so on [9–12]. However, the U.S. food and drug administration (FDA) has warned that the Gd chelates tend to cause nephrogenic system fibrosis and cerebral deposition [13–15]. In addition, the *T*_1_ imaging capability of the commercial Gd chelates is not strong due to their small longitudinal relaxivity (*r*_1_, ~ 4 mM^−1^ s^−1^) [16].

In order to overcome the problems of Gd chelates, increasing studies have been focusing on magnetic iron oxide nanoparticles (MIONs) due to their excellent biocompatibility [17–20]. Actually, MIONs were first used as *T*_2_-weighted CAs for examination of human liver in 1994 [21]. Several types of MIONs, such as Supravist, Feridex, and Rsovist, were developed and used as *T*_2_ CAs for MRI of human diseases in the 2000s [22]. However, these MION agents are not used in clinic anymore due to the following problems. (1) The MION agents produce darker MR images that are not conducive to the clinician’s diagnosis for diseases [23]. (2) Slow body clearance and long blood circulation lead to long waiting time for patients. (3) The high magnetic moment of MIONs can result in susceptibility artifacts. (4) The long echo time (TE) and repetition time (TR) result in long processing time of clinical MRI examinations. (5) Eovist, a liver-specific *T*_1_ contrast agent, was approved in 2008, and used to replace the MIONs-based *T*_2_ CAs.

Because there are no ideal products in clinic, MRI CAs have been one of the research hotspots for a long time. The recently emerging ES-MIONs (< 5.0 nm) with high *r*_1_ and low transversal relaxivity (*r*_2_) can be used as *T*_1_ CAs without concerns of nephrotoxicity and cerebral deposition [24–26]. Therefore, ES-MIONs can surmount drawbacks of the above-mentioned Gd chelates and MIONs. Kim et al. reported uniform ES-MIONs prepared by a method of thermal decomposition in 2011, which has low *r*_2_ value [27]. However, the ES-MIONs synthesized in oil phase are not soluble in water and need further hydrophilic functionalization on their surfaces, which severely limits their clinical applications. To solve this problem, we previously synthesized ES-MIONs with stabilization of poly (acrylic aid) (PAA) in aqueous phase by co-precipitation method [23]. The synthesized ES-MIONs can be easily dispersed in water, and the dispersion can be kept at room temperature for several months without any precipitation. However, the used stabilizer PAA is not biodegradable in human physiological environment.

**Scheme 1:**
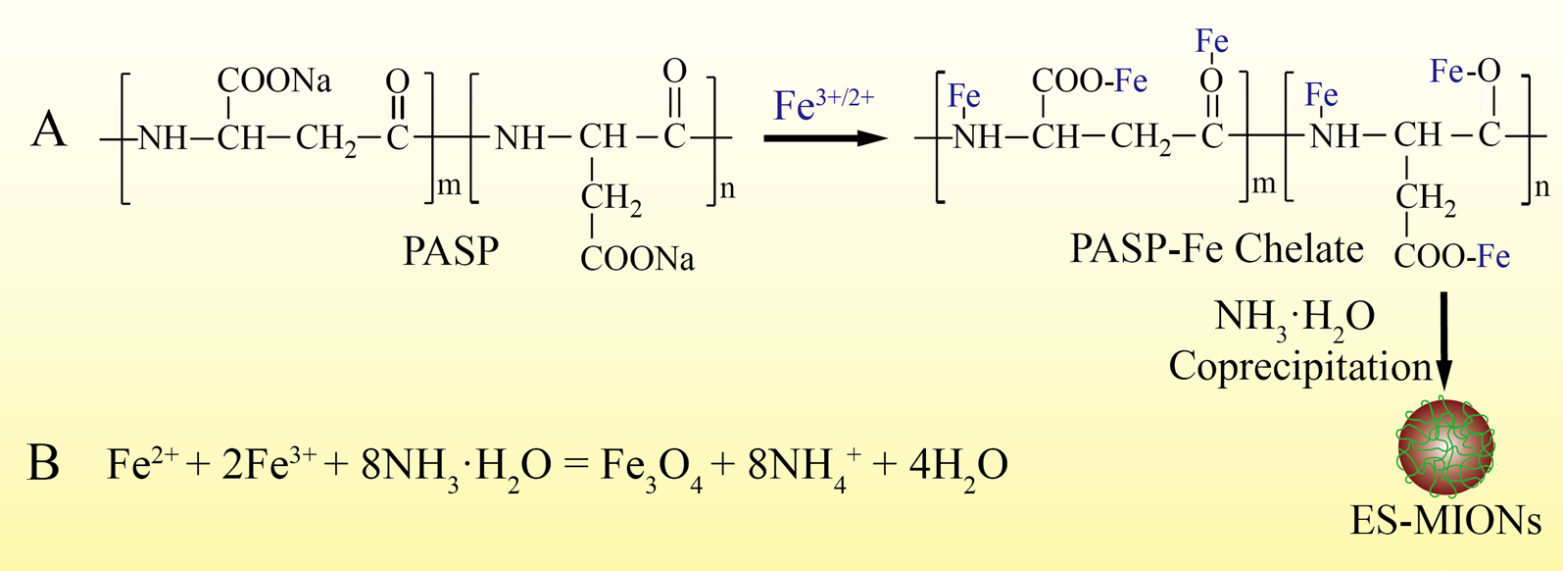
Schematic illustration of synthesis process (**A**) and reaction equation (**B**) for the ES-MIONs

In this study, a facile co-precipitation method was developed to synthesize biodegradable and biocompatible ES-MIONs (< 5.0 nm) with excellent water-dispersibility for *T*_1_-weighted MRI of tumors. As shown in Scheme 1A, biodegradable poly (aspartic acid) (sodium salt, PASP) first react with Fe^3+^ and Fe^2+^ to form PASP-Fe chelate, which can further react with ammonia solution producing biodegradable and biocompatible ES-MIONs via co-precipitation. The reaction equation generating Fe_3_O_4_ is shown in Scheme 1B. Due to the enrichment of carboxyl groups in the surface, the negatively charged ES-MIONs have excellent water-dispersibility. Because the amide bonds of PASP are biodegradable in human physiological environment, PASP can be used as an excellent candidate as the stabilizer for the synthesis of ES-MIONs. The PASP cannot be replaced with other poly(amino acids) because they are either less water-soluble than PASP, or positively charged. Because the *r*_1_ value (7.0 mM^−1^ s^−1^) is much higher than that of commercial Gd chelates (~ 4 mM^−1^ s^−1^) and the iron is one of the essential elements in the human body, the obtained ES-MIONs are biocompatible and have huge potential to be used as *T*_1_ MRI CAs, surpassing the commercial Gd chelates.

## Results and discussion

### Synthesis and characterization of ES-MIONs

**Fig. 1:**
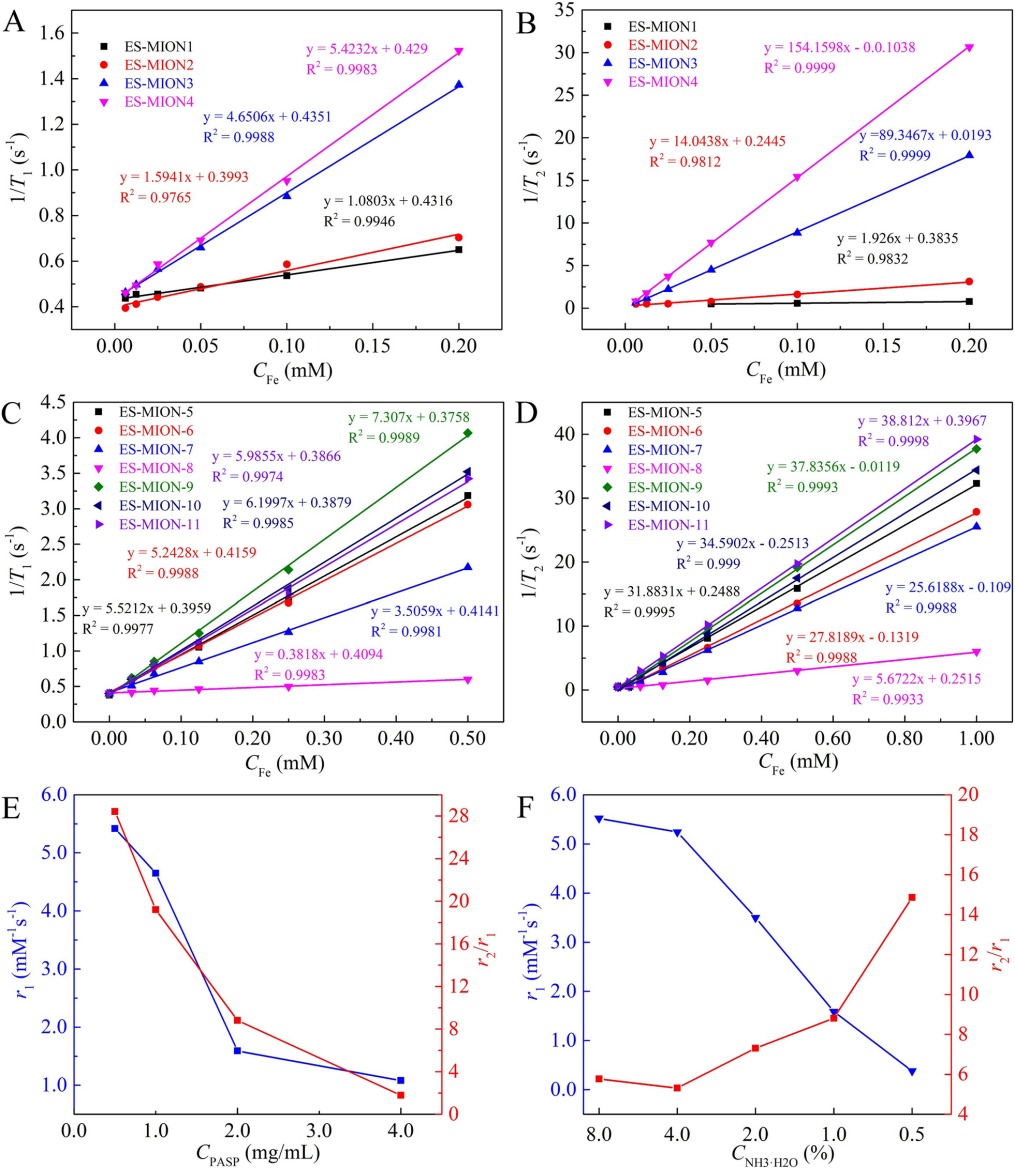
**A**–**D** *T*_1_ relaxation rate (1/*T*_1_) (**A**, **C**) or *T*_2_ relaxation rate (1/*T*_2_) (**B**, **D**) plotted versus *C*_Fe_ for ES-MION1-11. **E**, **F** The *r*_1_ or *r*_2_/*r*_1_ of the ES-MION1-4 (**E**) or ES-MION5-8 (**F**) as a function of *C*_PASP_ or *C*_NH3·H2O_. The magnetic field is 3.0 T

The ES-MIONs were synthesized by a method of co-precipitation, and reaction conditions were optimized to obtain high quality ES-MIONs with high *r*_1_ and *r*_2_/*r*_1_ (Additional file 1: Table S1). PASP was used as a stabilizer for the ES-MIONs preparation, which gives the obtained ES-MIONs excellent water dispersibility. Four concentrations of PASP solutions were used for synthesis of ES-MION1-4. The Fe concentration of ES-MIONs was determined by inductively coupled plasma-optical emission spectrometry (ICP-OES), and the ES-MION2 has the largest Fe recovery of 96.6% (Additional file 1: Table S1). *T*_1_ and *T*_*2*_ relaxation rates (3.0 T) versus Fe concentration of ES-MION1-4 are shown in Fig. 1A, B. The *r*_1_ and *r*_2_ values are obtained from the linear line slopes, which are summarized in Fig. 1E and Additional file 1: Table S1. The ES-MION2 has a *r*_1_ value of 1.6 mM^−1^ s^−1^ and *r*_2_/*r*_1_ ratio of 8.8. Though the *r*_1_ of ES-MION3, 4 is larger than ES-MION2, the *r*_2_/*r*_1_ values of ES-MION3, 4 are also much higher than that of ES-MION2, which are not good for T_1_ imaging. The *r*_2_/*r*_1_ value of ES-MION1 is lower than that of ES-MION2, but the *r*_1_ value is also lower than that of ES-MION2. Therefore, 2.0 mg/mL of PASP solution was considered as the optimal concentration for the synthesis of ES-MIONs.

Furthermore, 0.5–8.0% of ammonia solutions were used to synthesize ES-MION5-8, whose *T*_1_ and *T*_2_ relaxation rates (3.0 T) as a function of Fe concentration are shown in Fig. 1C, D. As shown in Fig. 1F and Additional file 1: Table S1, the *r*_1_ and *r*_2_/*r*_1_ of ES-MION6 are comparable to those of ES-MION5, but much better than ES-MION 7, 8. Therefore, 4.0% of ammonia solution was chosen as the optimal condition.

In addition, based on the optimized conditions for ES-MION6 synthesis, the concentration of PASP and iron precursors (FeCl_3_ plus FeSO_4_) were all decreased to synthesize ES-MION9-11. From Fig. 1C, D, F and Additional file 1: Table S1, it can be found that ES-MION9 has a highest *r*_1_ value of 7.0 ± 0.4 mM^−1^ s^−1^ (3.0 T) and a lowest *r*_2_/*r*_1_ value of 4.9 ± 0.6 (3.0 T) compared with ES-MION6, 10, 11. According to Eq. (1) [28], the signal intensity of MRI is depended on gradient intensity (M_0_), echo time (TE), repetition time (TR), flip Angle (α), R_2_^*^ and R_1_. The factors of M_0_, TE, TR, and α could be regulated by MRI scanners, while R_2_^*^ and R_1_ depend on contrast agents. The R_2_^*^ can be considered a valid R_2_ and is always greater than or equal to R_2_. It can be concluded that the T_1_ MRI signal intensity is proportional to *r*_1_ value, but inversely proportional to *r*_2_/*r*_1_ ratio. Thus, the synthesis conditions of ES-MION9 should be optimal to obtain a high T_1_ MRI capability with a high *r*_1_ and low *r*_2_/*r*_1_.

1 $${\text{Signal intensity = M}}_{{0}} {\text{sin(}}\alpha {)}\frac{{1 - {\text{e}}^{{ - {\text{R}}_{{1}} \cdot {\text{TR}}}} }}{{1 - {\text{cos(}}\alpha {)} \cdot {\text{e}}^{{ - {\text{R}}_{{1}} \cdot {\text{TR}}}} }}{\text{e}}^{{ - {\text{R}}_{{2}}^{*} \cdot {\text{TE}}}}$$

Besides, Fe recoveries of ES-MION1-11 tested by ICP-OES are all above 85%, indicating high utilization rates of raw materials and low cost for ES-MIONs synthesis, which are beneficial for clinical transformation.

**Fig. 2:**
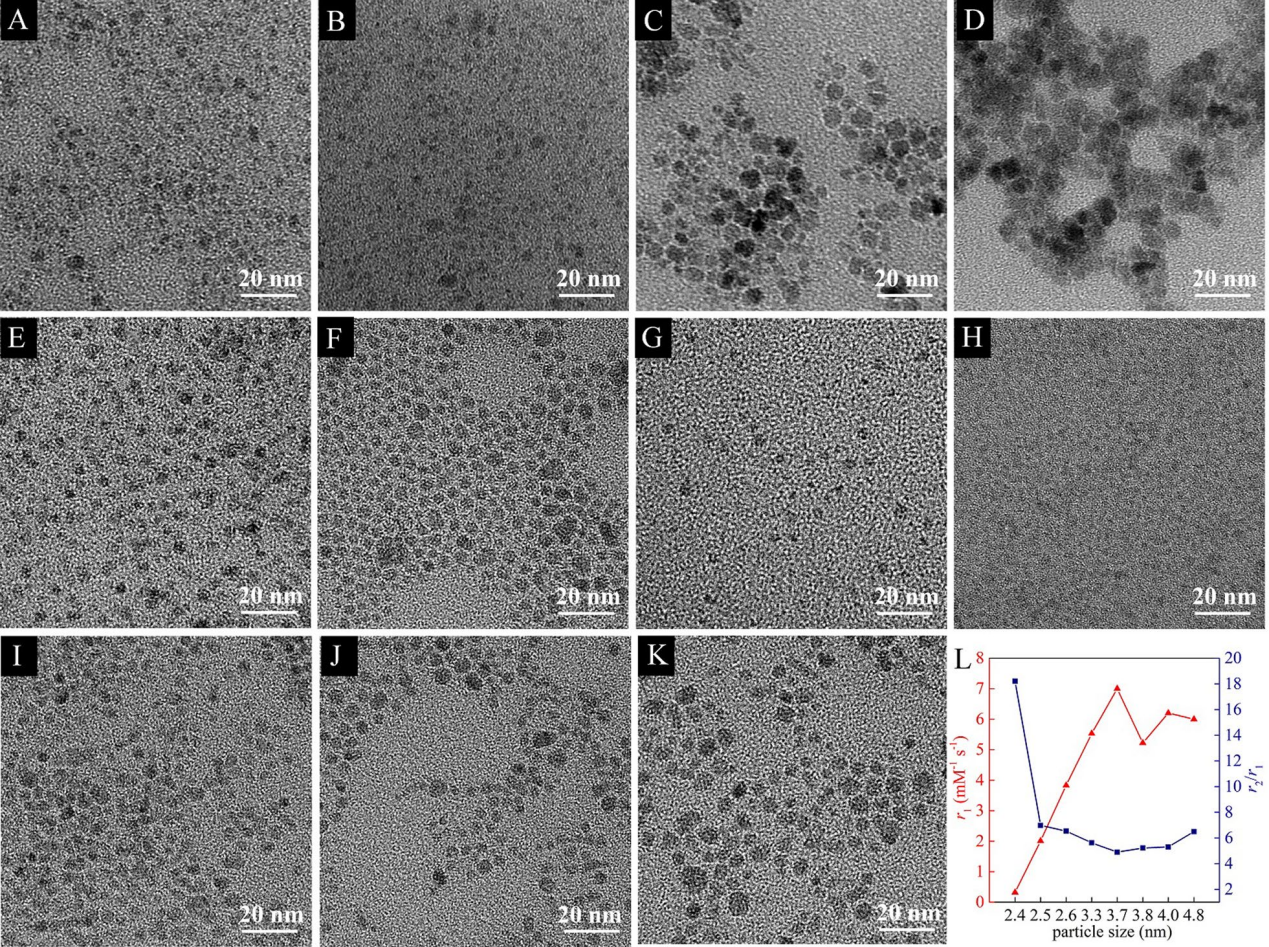
**A**–**K** TEM images of ES-MION1-11. **L** The *r*_1_ and *r*_2_/*r*_1_ of ES-MIONs plotted versus its diameter

According to previous reports, Fe_3_O_4_ nanoparticles with size below 5.0 nm can be used as *T*_1_ CAs [24]. Furthermore, Fe_3_O_4_ nanoparticles with large particle size are easily taken up by the spleen and liver, which seriously affects tumor images. The images of transmission electron microscopy (TEM, Fig. 2A–K) indicate our ES-MION1-11 have excellent water dispersibility. It is found from the TEM images (Fig. 2A–D) and size distributions (Additional file 1: Fig. S1A–D) measured from TEM images that the concentration of PASP has a large influence on the sizes of ES-MIONs. The sizes of ES-MION1-4 are respectively 2.7, 2.5, 6.0 and 8.0 nm, whose *r*_1_ is 1.0, 2.0, 4.7, and 5.4 mM^−1^ s^−1^, and the *r*_2_/*r*_1_ is 1.9, 7.0, 19.0, and 28.3. These results demonstrate that Fe_3_O_4_ nanoparticles with size below 5.0 nm have potential as *T*_1_ CAs, while those with size larger than 5.0 nm can be only utilized as *T*_2_ CAs due to the high *r*_2_/*r*_1_ ratios. Figure 2E–K and Additional file 1: Fig. S1E–K show that both the concentration of ammonia solution and the whole concentrations of feeding materials have a slight influence on the size of ES-MIONs. The relationships between the particle size and *r*_1_ value (or *r*_2_/*r*_1_ ratio) (Fig. 2L) show that the best particle size is 3.7 nm (ES-MION9).

Three batches of ES-MION9 were synthesized and the *T*_1_/*T*_2_ relaxation rates were determined by a 3.0 T (Additional file 1: Fig. S2) and 7.0 T MRI scanner (Additional file 1: Fig. S3), whose similar *r*_1_ and *r*_2_ data for different batches demonstrate the good repeatability for ES-MION9 synthesis. At 3.0 T, the ES-MION9 has a larger *r*_1_ (7.0 ± 0.4 mM^−1^ s^−1^) than Gadavist (4.9 ± 0.1 mM^−1^ s^−1^), indicating a stronger T_1_ MRI capability of our ES-MION9.

The related *T*_1_-weighted MR images (3.0 T) of ES-MION1-11 are shown in Additional file 1: Figs. S4A, S5A, and S6A. The corresponding SNR and ΔSNR values were calculated according to Eqs. (2) and (3) [29, 30], and shown in Additional file 1: Figs. S4B, S5B, and S6B, which reinforce that the signal intensities of MR images increase with the increase of Fe concentration with a strong concentration gradient dependence, showing good *T*_1_-weighted MR capabilities of ES-MION1-11.

2 $${\text{SNR}} = \frac{{{\text{SI}}_{{{\text{mean}}}} }}{{{\text{SD}}_{{{\text{noise}}}} }}$$

3 $$\Delta {\text{SNR}} = \frac{{({\text{SNR}}_{{{\text{sample}}}} - {\text{SNR}}_{{{\text{control}}}} )}}{{{\text{SNR}}_{{{\text{control}}}} }} \times 100\%$$

It is obvious that the ΔSNR value of ES-MION9 is the maximum up to 5500% when the Fe concentration of is 1.0 mM (Additional file 1: Fig. S6B), which further demonstrate 3.7 nm is the best diameter of ES-MIONs for *T*_1_ MRI.

**Fig. 3:**
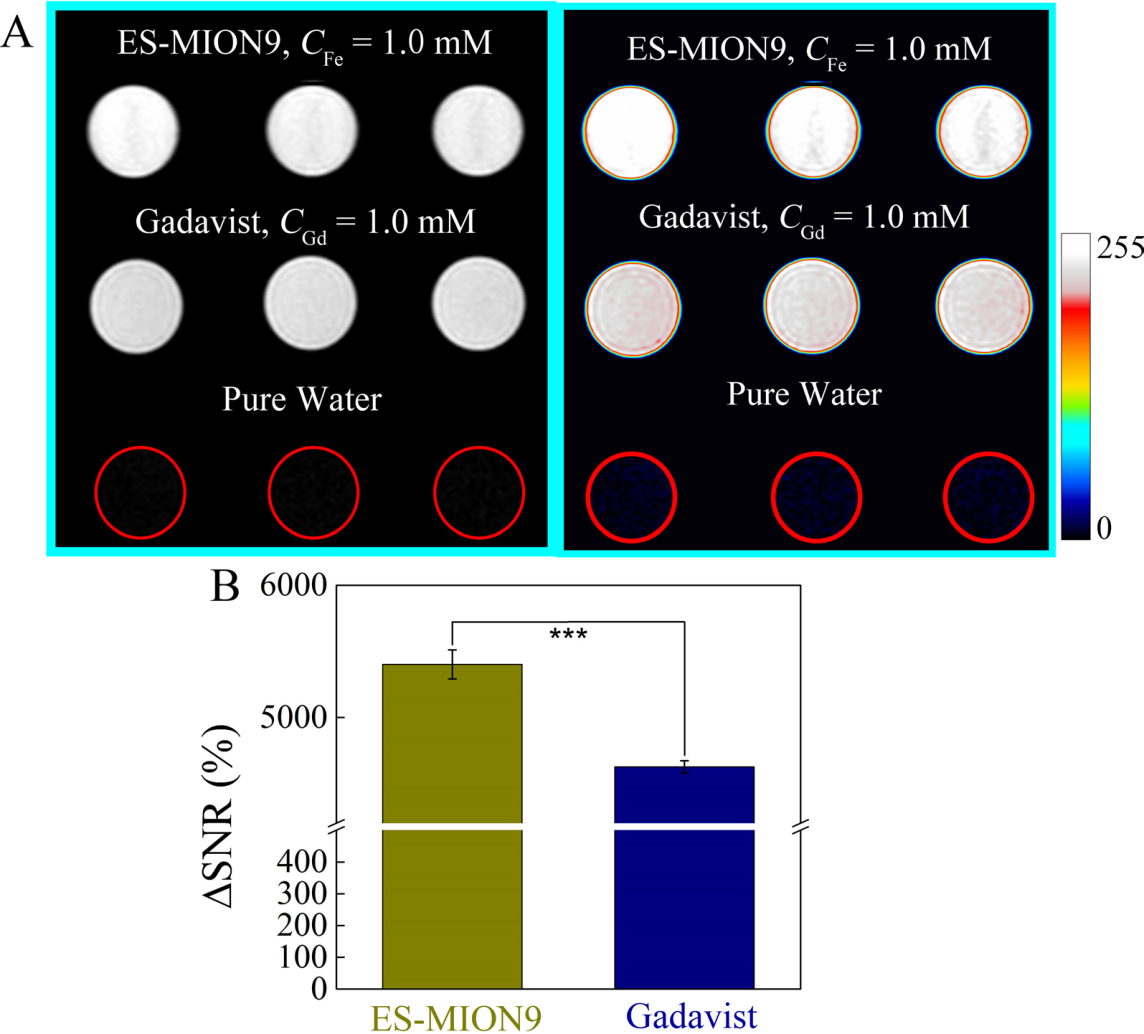
**A** *T*_1_-weighted MR images of ES-MION9 solutions (*C*_Fe_ = 1.0 mM) and commercial Gadavist solutions (*C*_Gd_ = 1.0 mM) compared with pure water (control). Magnetic field = 3.0 T. TE = 8.3 ms, TR = 200 ms. **B** ΔSNR of the MR images of ES-MION9 and Gadavist solutions, which is measured by the Image J. ***P < 0.001

The *T*_1_ images (3.0 T) of ES-MION9 solution at 1.0 mM were further compared with the commercial Gadavist at 1.0 mM of Gd concentration (Fig. 3A). It can be seen from Fig. 3B that the ΔSNR (5400%) of ES-MION9 is higher than that (4600%) of Gadavist (***P < 0.001), which demonstrates the better MR imaging capability of our ES-MION9 (*r*_1_ is 7.0 mM^−1^ s^−1^, *r*_2_/*r*_1_ is 4.9, 3.0 T) compared with the Gadavist.

A 7.0 T of MRI scanner was also used to double confirm the *T*_1_-weighted MRI contrast of ES-MION9 solutions at various concentrations compared with pure water (Additional file 1: Fig. S7A). The corresponding ΔSNR values (Additional file 1: Fig. S7B) also show a strong concentration gradient dependence, indicating a strong MRI capability at 7.0 T.

The ES-MION9 HR-TEM image is presented in Additional file 1: Fig. S8A. The lattice planes of 311 and 220 can be confirmed by the 0.51 and 0.301 nm of interplanar distances [31], indicating a crystalline structure of ES-MION9. The characteristic peaks of O and Fe can be found in the EDS (Additional file 1: Fig. S8B), demonstrating the component of iron oxide for ES-MION9 [32]. To further demonstrate the successful synthesis of Fe_3_O_4_ nanoparticles, the X-ray photoelectron spectroscopy (XPS) of ES-MION9 is performed in Additional file 1: Fig. S8C. The primary peaks at 723.8 and 710.3 eV correspond to the energy of Fe 2p3/2 and Fe 2p1/2 [33, 34], indicating the Fe_3_O_4_ component of our ES-MION9 [23]. Additional file 1: Fig. S8D shows the XRD of ES-MION9. Four characteristic peaks (2θ ≈ 30.0°, 35.2°, 42.8°, and 53.0°) match with the indices [(220), (311), (400), and (511)]. The crystal structure of ES-MION9 matches the pristine of Fe_3_O_4_, demonstrating the high crystalline purity of our ES-MION9. The field dependent magnetization curve (Additional file 1: Fig. S8E) indicates the ES-MION9 is superparamagnetic with 16.0 emu/g of saturation magnetization (M_s_). All these results indicate that the ES-MION9 we synthesized is superparamagnetic Fe_3_O_4_ nanocrystals.

Because the M_s_ values of ES-MIONs increase with the increasing particle sizes [28], the small M_s_ value of ES-MION9 indicates its small particle size. In Eq. (4), the *r* is the magnetic core radius and M_s_ is the saturation magnetization. According to Eq. (4), both the extremely small particle size (3.7 nm) and small M_s_ (16.0 emu/g) lead to a very low *r*_2_, which results in a very low *r*_2_/*r*_1_. Therefore, our exceedingly small ES-MION9 can be used as *T*_1_ CA.

4 $$\frac{1}{{{\text{T}}_{2} }} = \frac{{(256\uppi ^{2}\upgamma ^{2} /405){\text{V}}^{*} {\text{M}}_{{\text{S}}}^{2} {\text{r}}^{2} }}{{{\text{D}}(1 + {\text{L}}/{\text{r}})}}$$

The high *r*_1_ value of ES-MION9 is mainly due to the following two reasons: (1) ES-MION9 has a small particle size (3.7 nm), which gives ES-MION9 a larger specific surface area. In accordance with the mechanism of inner-sphere, larger specific surface area means there are more naked iron on ES-MION9 surfaces, which can fully interacts with hydrogen protons in H_2_O molecules, resulting in a high *r*_1_ value. (2) There are excessive carboxyl groups on ES-MION9 surfaces, and these carboxyl groups are derived from PASP, which greatly improves the water dispersion of ES-MION9. This leads to more H_2_O in the inner sphere that can interact with the naked iron on the ES-MION9 surface, which causes a large number of bound H_2_O (*q*) and mole fraction of H_2_O coordinated to Fe (*P*_m_) in Eq. (5) [16]. The large *q* and *P*_m_ result in a large *r*_1_ value for ES-MION9.

5 $$\frac{1}{{{\text{T}}_{1} }} = \frac{{{\text{q P}}_{{\text{m}}} }}{{{\text{T}}_{{1{\text{m}}}} + \tau_{{\text{M}}} }}$$

The T_1_/T_2_ relaxation rate (1/T_1_ or 1/T_2_) is plotted versus concentration for contrast agents, and the r_1_ and r_2_ values are calculated from the slopes of the corresponding fitting lines. T_1_ CAs increase signal intensity of T_1_ images by shortening the longitudinal relaxation time (T_1_) of protons, which leads to high r_1_ values. The Fe_3_O_4_ nanoparticles with size below 5.0 nm have low M_s_ values causing low r_2_ values according to Eq. (4). Both high r_1_ and low r_2_ result in low r_2_/r_1_. Therefore, the 3.7 nm of ES-MION9 (< 5.0 nm) could be utilized for T_1_ MRI [35, 36].

The hydrodynamic size (d_h_) of ES-MION9 is 13.7 nm (Additional file 1: Fig. S9A), which is larger than renal filtration threshold (~ 8 nm). The slightly larger hydrodynamic diameter prolongs blood circulation time overcoming the limited MRI time window problem of commercial Gd chelates. The zeta potential of ES-MION9 was measured to be − 55.0 mV (Additional file 1: Fig. S9B), which is due to the presence of excessive carboxyl groups on the surface. Charge plays a key role in the behavior of intravenously injected nanoparticles and pharmacokinetics. For example, nanoparticles agglomerate under charge-mediated nonspecific binding to serum proteins. Sufficient negative charges can avoid the agglomeration of ES-MION9 while avoiding uptake of the nanoparticles by normal cells during blood circulation, resulting in more accumulated ES-MION9 in tumors. Additional file 1: Fig. S9C shows that the hydrodynamic diameter of ES-MION9 do not change significantly during storage in water, 10.0% FBS and 0.9% NaCl solution for 1 week, demonstrating the great stability of ES-MION9.

Additional file 1: Fig. S10 shows UV–vis absorption spectra for ES-MION1-11, which are similar with that of reported ES-MIONs stabilized with other polymers [23]. Additional file 1: Fig. S11 shows the FT-IR of PASP and ES-MIONN9. The stretching vibration peak of –CH_2_– at 1400.6 cm^−1^ can be seen from the FT-IR of PASP and ES-MION9, indicating the existence of PASP on the surface of ES-MION9 [37]. In addition, the stretching vibration peak of Fe–O at 604.5 cm^−1^ can be seen from the FT-IR of ES-MION9, but not in the FT-IR of PASP, indicating the existence of iron in ES-MION9. These results prove the successful synthesis of Fe_3_O_4_ [38]. Additional file 1: Fig. S12 presents the curves of thermogravimetric analysis (TGA) and differential thermogravimetry (DTG) for ES-MION9. As the temperature increases, the mass of ES-MION9 continues to decrease, and becomes stable at 37.8% of remaining mass. This is similar to 40.1% of Fe_3_O_4_ loading content for ES-MION9 measured by ICP. This result further demonstrates the existence of PASP on the ES-MION9 surface.

### Cellular uptake, cytotoxicity assay and T_1_-weighted imaging of cells

**Fig. 4:**
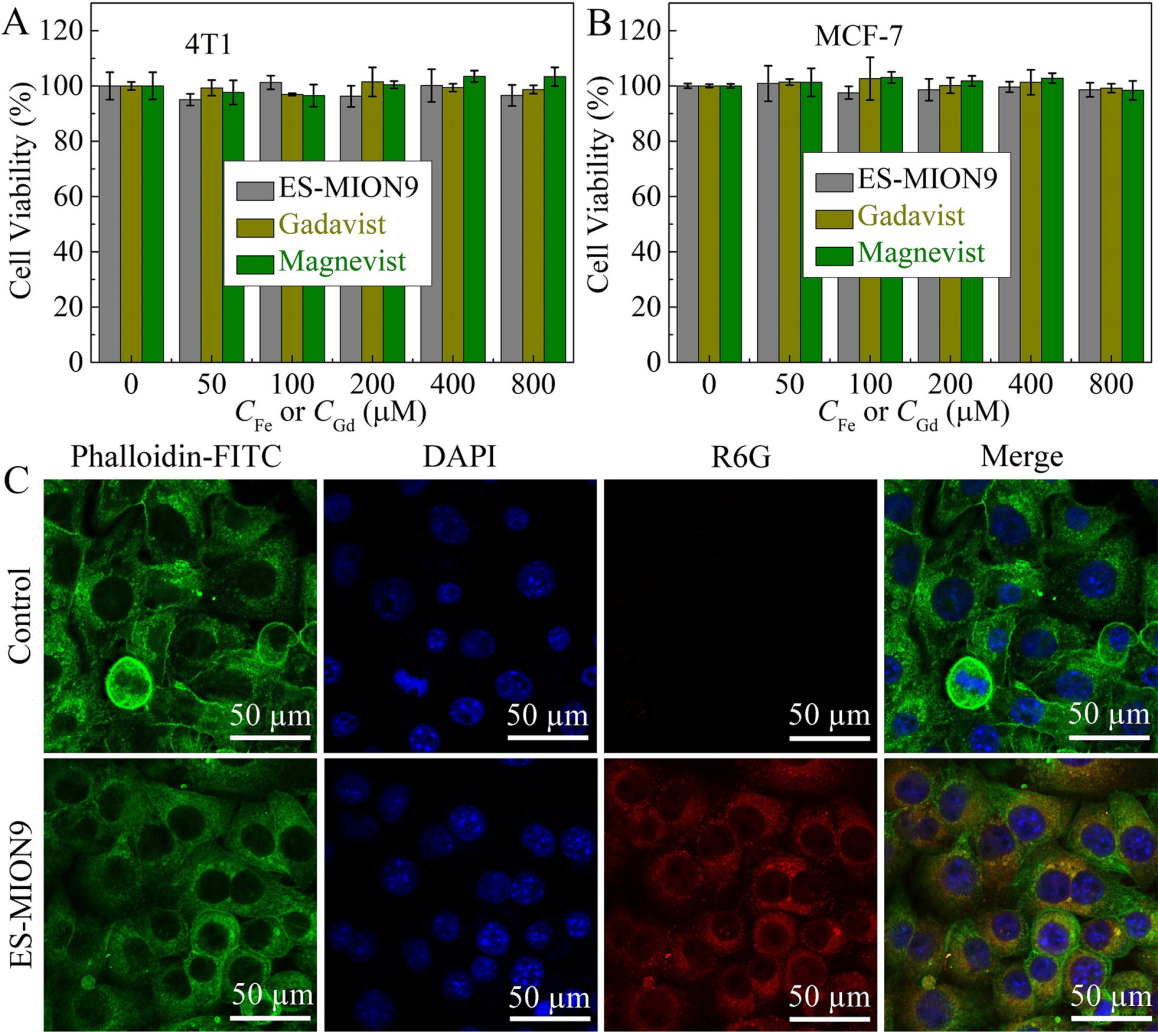
**A**, **B** Cytotoxicity of ES-MION9, commercial Gadavist or Magnevist on 4T1 cells or MCF-7 cells. Mean ± SD, n = 4. **C** LSCM images of 4T1 cells treated with ES-MION9@R6G for 2.0 h. The cytoskeleton is green due to the phalloidin-FITC staining, and the nucleus is blue due to the DAPI staining

To evaluate the biosafety of ES-MION9, its cytotoxicity was examined by thiazolyl blue tetrazolium bromide (MTT) assay on MCF-7 cells (Human breast cancer cells) and 4T1 cells (Mouse breast cancer cells). Figure 4A, B shows that when the Fe concentration of ES-MION9 reaches 0.8 mM, the cell viability of MCF-7 cells and 4T1 cells was higher than 95.0%. This result indicates that ES-MION9 is almost not cytotoxic due to its biocompatible components (i.e., Fe_3_O_4_ and PASP). Although Gd^3+^ can cause nephrogenic systemic fibrosis and can be deposited in the human brain and body [39], Fig. 4A, B shows that the Gadavist is also non-toxic at the Gd concentration of 0.8 mM. That’s because Gd^3+^ leads to long-term toxicity, which cannot be revealed in the short-term MTT assay.

To further demonstrate the non-cytotoxicity of ES-MION9, live/dead cytotoxicity analysis was used to evaluate the toxicity of ES-MION9 to 4T1 cells and MCF-7 cells (Additional file 1: Figs. S13, S14). The PBS treated cells were used as a control. Green dots represent live cells and red dots represent dead cells. Obviously, almost no dead cells are found for ES-MION9-treated 4T1 cells and MCF-7 cells, showing good biosafety of ES-MION9. That’s because the main component aspartic acid (ASP) is one of the 20 essential amino acids and iron is one of the essential elements in the human body.

Figure 4C shows the LSCM images of 4T1 cells treated with ES-MION9@R6G. The red signal represents R6G@ES-MION9. After 2 h of co-incubation with 4T1 cells, lots of ES-MION9 nanoparticles were found inside the cells (Fig. 4C). The uptake of ES-MION9 by 4T1 cells was further investigated by flow cytometry. After 2 h of co-incubation with 4T1 cells, the fluorescence intensity (Additional file 1: Fig. S15A, B) of R6G-labeled ES-MION9 was almost two orders of magnitude higher than that of the control group with a statistical P value smaller than 0.001, indicating that ES-MION9 is easily taken up by 4T1 cells. The results of flow cytometry are consistent with the LSCM results. In addition, the *T*_1_-weighted MR images (7.0 T) (Additional file 1: Fig. S16) show that ES-MION9-treated tumor cells have much stronger MRI signals compared to the control groups, and the MR signal also increases with the increase of incubation time from 1.0 to 2.0 h. These results demonstrate the excellent MR imaging capability of our ES-MION9 at the cellular level.

### In vivo MR imaging

**Fig. 5:**
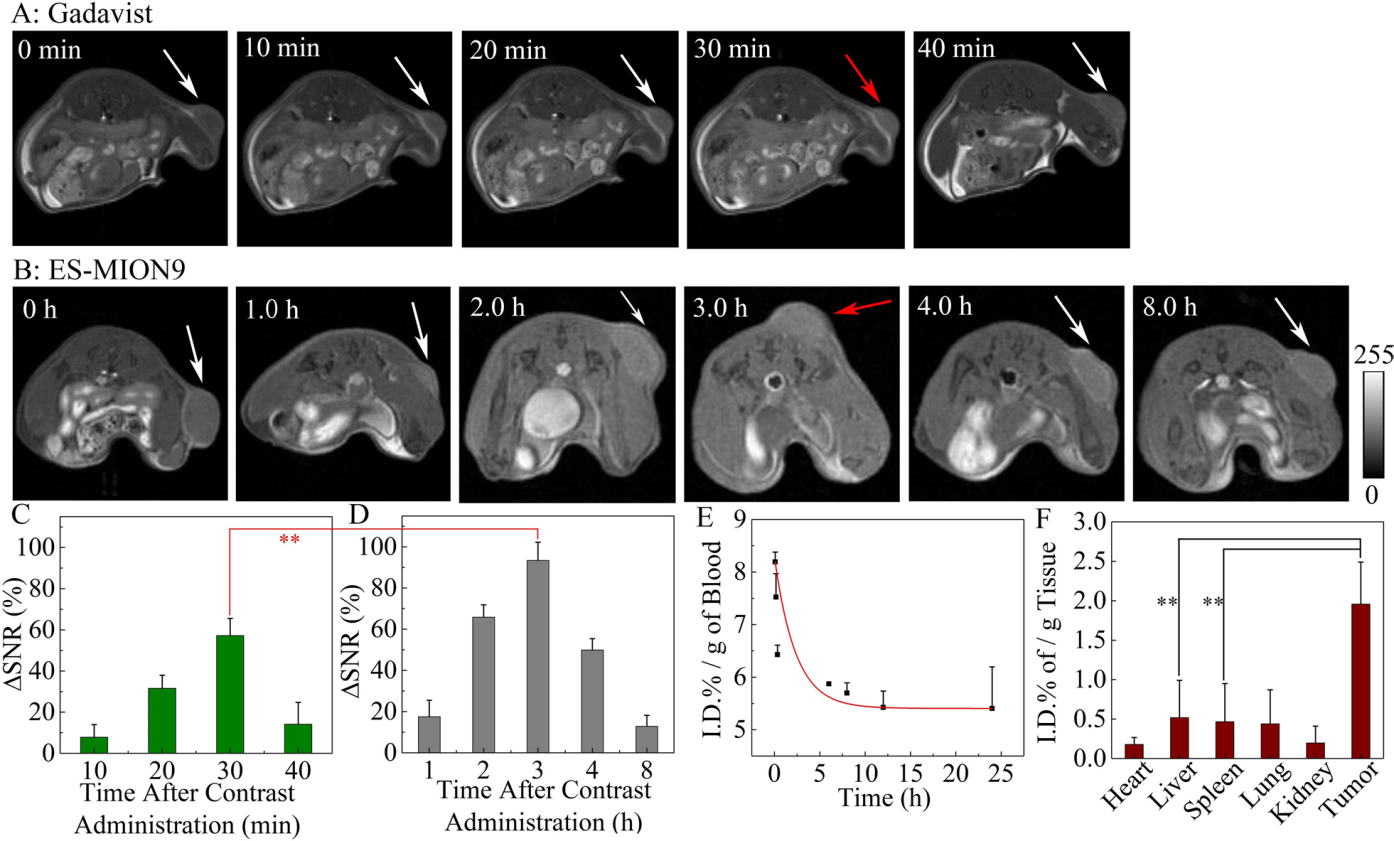
**A**, **B**: *T*_1_-weighted MR images of 4T1 tumor-bearing BALB/c mice with or without i.v. injection of Gadavist at 5.0 mg/kg of Gd dosage (**A**), or ES-MION9 at 5.0 mg/kg Fe dosage (**B**) under 7.0 T of magnetic field. **C**, **D** ΔSNR of the MR images for Gadavist (**C**), or ES-MION9 (**D**). **E**, **F** Blood clearance profile (**E**) and in vivo biodistribution of Fe level (**F**) in the 4T1 tumor-bearing BALB/c mice after i.v. injection of ES-MION9. Fe dosage is 5.0 mg/kg. **P < 0.01

MRI can be used for soft tissue imaging, especially for tumor diagnosis. MR contrast agents can improve the signal-to-noise ratio and sensitivity of MRI. We tested the imaging ability of ES-MION9 in 4T1 tumor-bearing mice. 4T1 cells were seeded subcutaneously into BALB/c mice to build 4T1 tumor models. The commercial Gadavist and our ES-MION9 were i.v. injected into the 4T1 tumor-bearing mice for MR imaging (Fig. 5A, B). It can be seen from the MR images that after the administration of Gadavist or ES-MION9, the tumor becomes brighter than that of control (pre-injection), and reaches the brightest at 30 min or 3.0 h post-injection, respectively. MR images of different slices were obtained at each time point, and the brightest one of different slices at each time point was selected to characterize the MR imaging capabilities. Because the contrast difference between tumor and normal tissue is usually hard to be identified by the naked eyes, the signal changes in tumors at various time points after the administration of contrast agents are quantified using ΔSNR as shown in Fig. 5C, D, which is calculated according to the Eq. (6):

6 $$\Delta {\text{SNR}} = \frac{{({\text{SNR}}_{{{\text{post}}}} - {\text{SNR}}_{{{\text{pre}}}} )}}{{{\text{SNR}}_{{{\text{pre}}}} }} \times 100\%$$

The ΔSNR value is up to 93.4% at 3.0 h after administration of ES-MION9 (Fig. 5D), which is significantly larger than that of the tumor at 30 min post-injection of Gadavist (57.2%, Fig. 5C). The above results demonstrate that our ES-MION9 can be utilized as a stronger MRI CAs compared with the clinically used Gd chelates.

### Pharmacokinetics, biodistribution and biosafety evaluation in vivo

To verify that our ES-MION9 is more biocompatible and safer than Gadavist, the pharmacokinetics, biodistribution and biosafety were evaluated in vivo. Figure 5E shows that the blood half-life of ES-MION9 is about 2.3 h due to the small nanoparticle size (3.7 nm). The best time window for MRI in clinic is close to the half-life (10–15 min) of commercial Gd chelates, which is a little bit tight for MRI after administration of the Gd chelates [40]. The slightly longer half-life of our ES-MION9 overcomes the limited MRI time window problem of commercial Gd chelates.

To evaluate the biodistribution of ES-MION9 in vivo, the Fe contents in the heart, liver, spleen, lung, kidney and tumor of mice were measured at 0 h pre-injection and 12.0 h post-injection of ES-MION9, and the differences are shown in Fig. 5F. It is found the ES-MION9 accumulation inside tumors is very high compared with other normal tissues because of the enhanced permeability and retention (EPR) effect, which is the key reason for the highly enhanced MRI signal of tumors after ES-MION9 injection.

Additional file 1: Fig. S17 shows the representative optical microscopic pictures of the H&E-stained main organs from the normal mice without tumors (control), or that with i.v. injection of ES-MION9 (*C*_Fe_ = 5.0 mg/kg). Compared with controls, ES-MION9-treated mice showed no obvious pathological abnormalities in major organs (heart, liver, spleen, lung, and kidney), indicating that our ES-MION9 does not lead to systemic toxicity.

## Conclusions

In summary, in order to surmount the problems of commercial Gd chelates-based *T*_1_ CAs, commercial MIONs-based *T*_2_ CAs, and reported ES-MIONs-based *T*_1_ CAs, a facile method based on co-precipitation was developed to synthesize biodegradable and biocompatible ES-MIONs with excellent water-dispersibility for *T*_1_ MRI of tumors using PASP as the stabilizer. After optimization of the synthesis conditions, the final obtained ES-MION9 with a diameter of 3.7 nm has a high *r*_1_ (7.0 ± 0.4 mM^−1^ s^−1^) and a low *r*_2_/*r*_1_ (4.9 ± 0.6) at 3.0 T. The ES-MION9 has excellent water dispersibility due to the excessive carboxyl groups from PASP. The physical properties of ES-MION9 were further characterized by TEM, XRD, EDS, XPS, UV–vis, FT-IR, TGA, and magnetization curve. LSCM images and flow cytometry results prove the cellular uptake of ES-MION9 by endocytosis. The pharmacokinetics, and biodistribution of ES-MION9 in vivo demonstrate the better tumor targetability and MRI time window of ES-MION9 than commercial Gd chelates. *T*_1_-weighted MR images of aqueous solutions, cells and tumor-bearing mice at 3.0 T or 7.0 T demonstrate that our ES-MION9 has a stronger MRI capability than the commercial Gd chelates. The MTT assay, live/dead staining of cells, and H&E-staining indicate the non-toxicity and biosafety of our ES-MION9. Consequently, the biodegradable and biocompatible ES-MION9 with excellent water-dispersibility is an ideal *T*_1_-weighted CAs with promising translational possibility to compete with the commercial Gd chelates.

## Materials and methods

### Synthesis of ES-MIONs

In order to eliminate O_2_, 20.0 mL and 0.5–4.0 mg mL^−1^ of PASP (M_w_ = 7000) solution was first bubbled using N_2_ for 60 min. After that, the solution was heated to 100 °C under reflux. A Fe solution (0.4 mL, 125.0–500.0 mM FeCl_3_ + 62.5–250.0 mM FeSO_4_) was then rapidly charged to the above-mentioned PASP solution. Subsequently, NH_3_^**.**^H_2_O (6.0 mL, 0.5–8.0%) was added under magnetic stirring. After 1.0 h, the reaction was stopped by cooling off. Finally, the synthesized ES-MIONs were purified via dialysis (Mw cut-off 8–14 kDa) in pure water for purification. An ICP-OES (iCAP PRO, Thermo Fisher Scientific, US) was used to determine the *C*_Fe_ of the ES-MIONs.

### Synthesis of R6G@ES-MION9

At room temperature, 70.0 µL of Rhodamine 6G (100.0 μM) was added into 4.0 mL of ES-MION9 (*C*_Fe_ = 2.8 mM), and the mixture was magnetically stirred for 24.0 h. The prepared R6G@ES-MION9 solution was then centrifugally ultra-filtrated (Millipore, Mw cutoff 10 kDa) and washed utilizing ultrapure water for purification. Finally, the obtained R6G@ES-MION9 was resolved in ultrapure water (4.0 mL) and kept in 4.0 °C of refrigerator.

## Supplementary Information

**Additional file 1. **The online version contains supplementary material available at https://jnanobiotechnology.biomedcentral.com.

## Data Availability

All data associated with this study are present in the paper and/or the additional file.
