# Supplementary material for: Biodegradable and biocompatible exceedingly small magnetic iron oxide nanoparticles for T1-weighted magnetic resonance imaging of tumors
Source: J Nanobiotechnology. 2022 Jul 30;20:350. doi: 10.1186/s12951-022-01562-y (PMC9338602; doi:10.1186/s12951-022-01562-y)
Supplement: Supplementary file 1 — Additional file 1. The online version contains supplementary material available at https://jnanobiotechnology.biomedcentral.com. [file 12951_2022_1562_MOESM1_ESM.docx]

Additional file

Biodegradable and Biocompatible Exceedingly Small Magnetic Iron Oxide Nanoparticles for T_1_-Weighted Magnetic Resonance Imaging of Tumors

Xuanyi Lu,^†,1^ Huimin Zhou,^‡,1^ Zhiyu Liang,^§^ Jie Feng,^§^ Yudie Lu,^†^ Lin Huang,^†^ Xiaozhong Qiu,*^,‡^ Yikai Xu,*^,§^ Zheyu Shen*^,†,‡,§^

^†^ Biomaterials Research Center, School of Biomedical Engineering, Southern Medical University, 1023 Shatai South Road, Guangzhou, Guangdong 510515, China.

^‡^ Guangdong Provincial Key Laboratory of Construction and Detection in Tissue Engineering, School of Basic Medical Sciences, Southern Medical University, 1023 Shatai South Road, Guangzhou, Guangdong 510515, China.

^§^ Medical Imaging Center, Nanfang Hospital, School of Biomedical Engineering, Southern Medical University, 1023 Shatai South Road, Guangzhou, Guangdong 510515, China.

**Corresponding Authors**

*E-mail: sz@smu.edu.cn (Z.S.), yikaivip@163.com (Y.X), qqiuxzh@163.com (X.Q.)

**Author Contributions**

^1^ X.L. and H.Z. contributed equally to this work.

**Materials and Methods**

**Materials**

Poly (aspartic acid) (sodium salt, PASP, M_w_ = 7000), ammonium hydroxide solution (NH_3_·H_2_O, 25-28%), iron (III) chloride (FeCl_3_, ≥99%) and iron (II) sulfate heptahydrate (FeSO_4_·7H_2_O, 99.95%), sodium hydroxide (NaOH, 97%), and Rhodamine 6G (R6G) were purchased from Maklin Reagent (Shanghai, China). 2-(4-Amidinophenyl)-6-indolecarbamidine dihydrochloride (DAPI) and FITC-Phalloidin were purchased from Beyotime Biotechnology (Shanghai, China). Calcein-AM/PI Solution was purchased from Dalian Meilun Biotech Co., Ltd. Thiazolyl blue tetrazolium bromide (MTT) was purchased from Shanghai Acmec Biochemical Co., Ltd.

**MRI Performance *In Vitro***

The MR imaging and relaxation times of the nanoparticles were tested by a Bruker MRI scanner (7.0 T, PharmaScan70/16, Bruker, US), and a clinical MRI scanner system (3.0 T, Ingenia, Philips, Netherlands), respectively. The relaxivity values of *r*_1_ and *r*_2_ were obtained as the slopes from the linear curves of relaxation rate (1/*T*_1_ or 1/*T*_2_, s^-1^) versus the Fe concentration (mM). Meanwhile, MR images were analyzed by measuring signal intensity with the software Image J. The signal-to-noise ratio (SNR) and ΔSNR value (*i.e.*, signal enhancement) were calculated according to the equation (1) and (2), respectively.

SNR = SI_mean_/SD_noise_ (1)

ΔSNR = (SNR_sample_ - SNR_control_)/SNR_control_ × 100 % (2)

**Cell Culture**

4T1 (mouse breast cancer cell line) and MCF-7 (human breast cancer cell line) cells were cultured in the complete DMEM medium supplemented with 10.0% of FBS and 1.0% of penicillin‐streptomycin solution (100×). All of the cells were incubated at 37°C in a humidified atmosphere containing 5.0 % of CO_2_.

**Cytotoxicity Assay**

The cytotoxicity of ES-MION9 were assessed on 4T1 or MCF-7 cells by MTT assay. Typically, 100 μL of MCF-7 or 4T1 cells in complete medium were seeded into each well of a 96-well plate at a concentration of 1.0×10^5^ cells/mL. After 24.0 h of culture, the medium was replaced with a fresh one without FBS, containing various concentrations of ES-MION9, Magnevist or Gadavist. After 2.0 h of incubation, the culture medium was replaced with a fresh complete medium, and the cells were further incubated for 24.0 h. After that, 10.0 μL of MTT solution (5.0 mg/mL in PBS) was added to each well of the 96-well plates. After an additional 4.0 h of incubation, the culture medium was removed and the resulted formazan crystals in each well were dissolved in 100 μL of dimethyl sulfoxide (DMSO) with shaking at room temperature for 20 min. The absorbance of each well at a wavelength of 490 nm was recorded on a multi-mode microplate reader (Synergy H1, BioTek Instruments, USA).

**Cellular Uptake**

Cellular uptake of nanoparticles were measured *via* laser scanning confocal microscopy (LSCM), Flow Cytometry, and magnetic resonance imaging (MRI).

By LSCM: 0.5 mL of 4T1 cells in complete medium were seeded in confocal dishes at a density of 1.0×10^5^ cells/mL and incubated for 24.0 h. The growth medium was replaced with a fresh one without or with R6G@ES-MION9 (*C*_Fe_ = 100.0 μM). After 2.0 h of incubation, the cells were washed twice with PBS, fixed with 4.0 % of formaldehyde for 30 min, permeabilized with 0.1 % of Triton X-100 for 5.0 min, blocked with 1.0 % of BSA for 30 min, and then stained with a mixture of Phalloidin-FITC and DAPI for 30 min at room temperature. After twice washes with PBS, the LSCM images of the samples were observed on a LSCM (Nikon ECLIPSE Ti2).

By flow cytometry: 2.0 mL of 4T1 or MCF-7 cells in growth medium were seeded into each well of a 6-well plate with a cell density of 2.0×10^5^ cells/mL. After 24 h of incubation, the growth medium was then replaced with a fresh one containing R6G@ES-MION9 (*C*_Fe_ = 100.0 μM). After further incubation for 2.0 h, the cells were washed twice with cold PBS, treated with trypsin for 3.0 min and then centrifuged at 800×g for 3.0 min. The obtained cells were resuspended in 0.5 mL of PBS and then measured by flow cytometer (BD FACSAria Ⅲ, USA).

By MRI: 4T1 or MCF-7 cells were seeded into 100 mm of cell culture dishes at a density of 1.5 million cells/dish and incubated for 72.0 h. The medium was then replaced with a fresh one containing ES-MION9 (*C*_Fe_ = 100.0 μM), and the cells were cultured 1.0, or 2.0 h. The cells were washed twice to remove free nanoparticles, and then treated with trypsin. After transferring into 200.0 µL of centrifuge tubes, the cells were centrifuged at 3200 × g for 5.0 min. The supernatant was removed and 0.2 mL of agarose aqueous solution (3.0 %) was added to fix the cells. *T*_1_-weighted MR images of the cells treated with nanoparticles were acquired using a Bruker MRI scanner (7.0 T, B-C 70/16, Bruker, US).

**Tumor Model**

All animal experiments were performed according to the Guidelines for Care and Use of Laboratory Animals of Southern Medical University, and approved by the Animal Ethics Committee of Southern Medical University. The 4T1 tumor-bearing BALB/c mice models were established by subcutaneously inoculating 4T1 cells (5 × 10^6^ cells in 100 μL PBS) onto the right back side of each mouse (female, 5 weeks) under anesthesia. The size of tumors was measured every other day with a vernier caliper, and the tumor volumes were calculated as follows: tumor volume (mm^3^) = width^2^ × length / 2.

***In Vivo* *T*_1_-weighted MRI**

The 4T1 tumor-bearing BALB/c mice were anaesthetized by isoflurane (1.0-2.0%) in oxygen, and placed in an animal-specific body coil for MRI data acquisition (7.0 T, PharmaScan70/16 US, Bruker, US). Mice were kept warm by circulating warm water (37℃), and were placed in a stretched prone position with a respiratory sensor during the experiments. *T*_1_-weighted MR images were acquired at pre- and post-injection (intravenously) of ES-MION9 (Fe dosage = 5.0 mg/kg) or Gadavist (Gd dosage = 5.0 mg/kg). Multi-slice multi-echo sequence was employed to acquire images using parameters as follows: repetition time (TR) = 200 ms, echo time (TE) = 7.3 ms, flip angle = 180º, matrix size = 256 × 256, field of view = 40 × 40 mm^2^, slices = 8, slice thickness = 1.5 mm. MR images were analyzed by measuring signal intensity with the software Image J. The signal-to-noise ratio (SNR) and ΔSNR values were calculated according to equations (3) and (4).

SNR = SI_mean_/SD_noise_ (3)

ΔSNR = (SNR_post_ - SNR_pre_)/SNR_pre_ × 100 % (4)

**Biodistribution of Nanoparticles**

To evaluate the biodistribution of nanoparticles *in vivo*, tumor-bearing BALB/c mice (n = 3) were injected with 100.0 μL of PBS or ES-MION9 solution (*C*_Fe_ = 5.0 mg/kg). The mice were sacrificed after 12.0 h, and then the major organs (heart, liver, spleen, lung, kidney) and tumors were collected. The Fe contents in all samples were determined by ICP-OES.

**Statistical Analysis**

All experimental data are presented as mean ± S.D. Statistical significance was determined by applying Student’s t-test or by a one-way ANOVA followed by Student-Newman-Keuls test using the software of SPSS 19.0. The significance level was fixed as * P < 0.05, ** P < 0.01, *** P < 0.001, **** P < 0.0001.

**Table S1.** Synthesis conditions and characterization results of the ES-MIONs.

| Sample  Nomenclature | *C*_PASP_ ^a^  (mg/mL) | *C*_FeCl3_ ^a^  (mM) | *C*_FeSO4_ ^a^  (mM) | *C*_NH3·H2O_ ^a^  (%) | Average  Particle  Size ^b^ (nm) | Fe Recovery ^c^ (%) | H_0_ (T) | *r*_1_ ^d^  (mM^-1^ s^-1^) | *r*_2_ ^d^  (mM^-1^ s^-1^) | *r*_2_/*r*_1_ |
| --- | --- | --- | --- | --- | --- | --- | --- | --- | --- | --- |
| ES-MION1 | 4.0 | 500 | 250 | 1.0 | 2.7 | 92.7 | 3.0 | 1.1 | 1.9 | 1.8 |
| ES-MION2 | 2.0 | 500 | 250 | 1.0 | 2.5 | 96.6 | 3.0 | 1.6 | 14.0 | 8.8 |
| ES-MION3 | 1.0 | 500 | 250 | 1.0 | 6.0 | 89.3 | 3.0 | 4.7 | 89.3 | 19.2 |
| ES-MION4 | 0.5 | 500 | 250 | 1.0 | 8.0 | 90.3 | 3.0 | 5.4 | 154.2 | 28.4 |
| ES-MION5 | 2.0 | 500 | 250 | 8.0 | 3.3 | 99.2 | 3.0 | 5.5 | 31.9 | 5.8 |
| ES-MION6 | 2.0 | 500 | 250 | 4.0 | 3.8 | 98.4 | 3.0 | 5.2 | 27.8 | 5.3 |
| ES-MION7 | 2.0 | 500 | 250 | 2.0 | 2.6 | 98.8 | 3.0 | 3.5 | 25.6 | 7.3 |
| ES-MION8 | 2.0 | 500 | 250 | 0.5 | 2.4 | 97.5 | 3.0 | 0.4 | 5.7 | 14.9 |
| ES-MION9 | 1.5 | 375 | 187.5 | 4.0 | 3.7 | 89.6 | 3.0 | 7.0±0.4 | 34.0±3.3 | 4.9±0.6 |
|  |  |  |  |  |  |  | 7.0 | 0.9±0.1 | 48.9±1.5 | 52.5±3.0 |
| ES-MION10 | 1.0 | 250 | 125.0 | 4.0 | 4.0 | 87.7 | 3.0 | 6.2 | 34.6 | 5.6 |
| ES-MION11 | 0.5 | 125 | 62.5 | 4.0 | 4.8 | 97.7 | 3.0 | 6.0 | 38.8 | 6.5 |
| Gadavist | - | - | - | - | - | - | 3.0 | 4.9±0.1 | 5.2±0.1 | 1.1±0.1 |

^a^ Concentration of the feeding materials before reaction.

^b^ Average diameter of the ES-MIONs measured by TEM.

^c^ Calculated from the molar ratio of Fe in the obtained ES-MIONs to that in the feeding materials (mean ± SD, *n* = 3).

^d^ Measured on a 7.0 T of MRI scanner, or a clinical MRI scanner (3.0 T). Mean ± SD, *n* = 3.


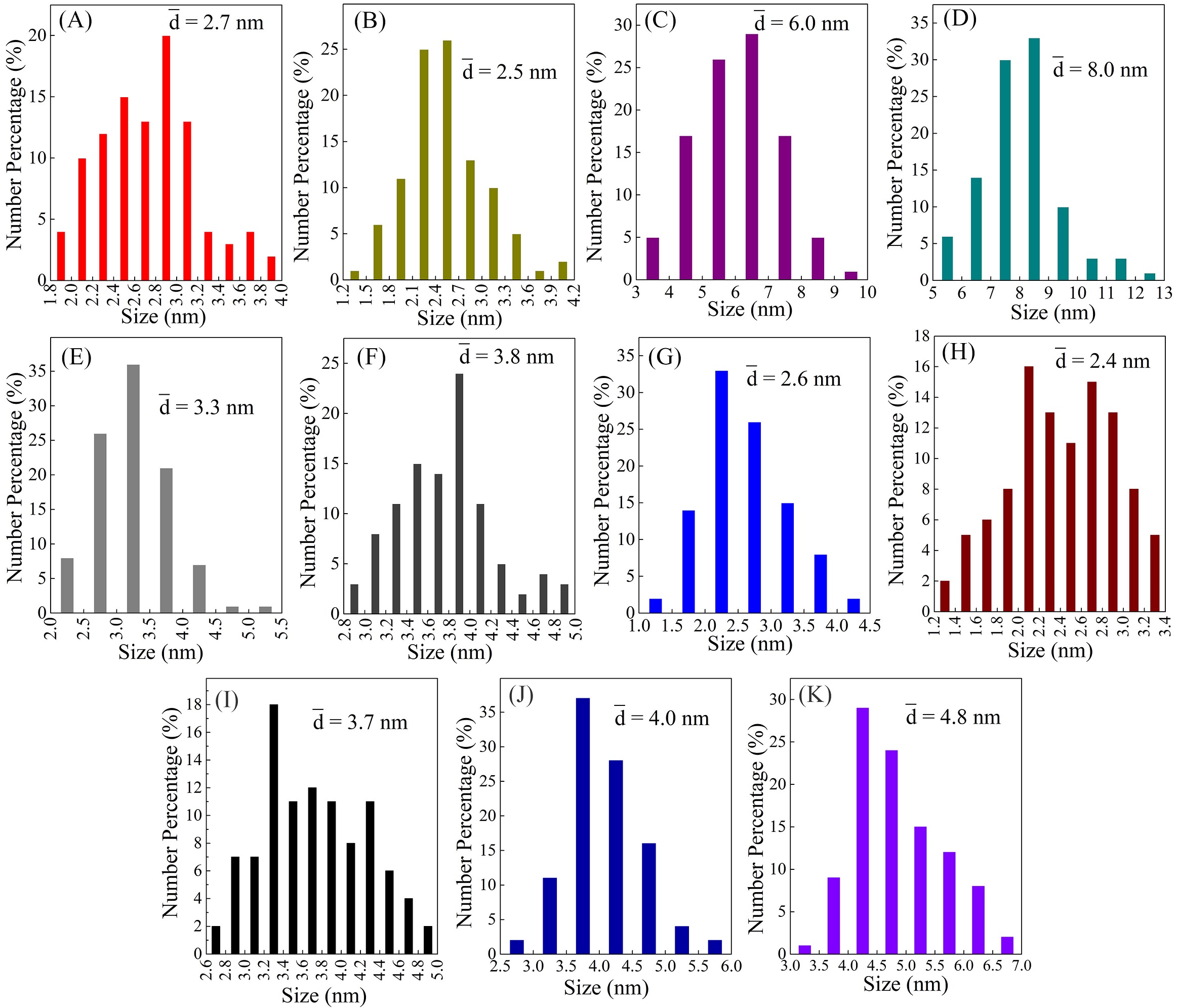


**Figure S1.** (A-K): Size distributions of ES-MION1-11 measured from the TEM images.


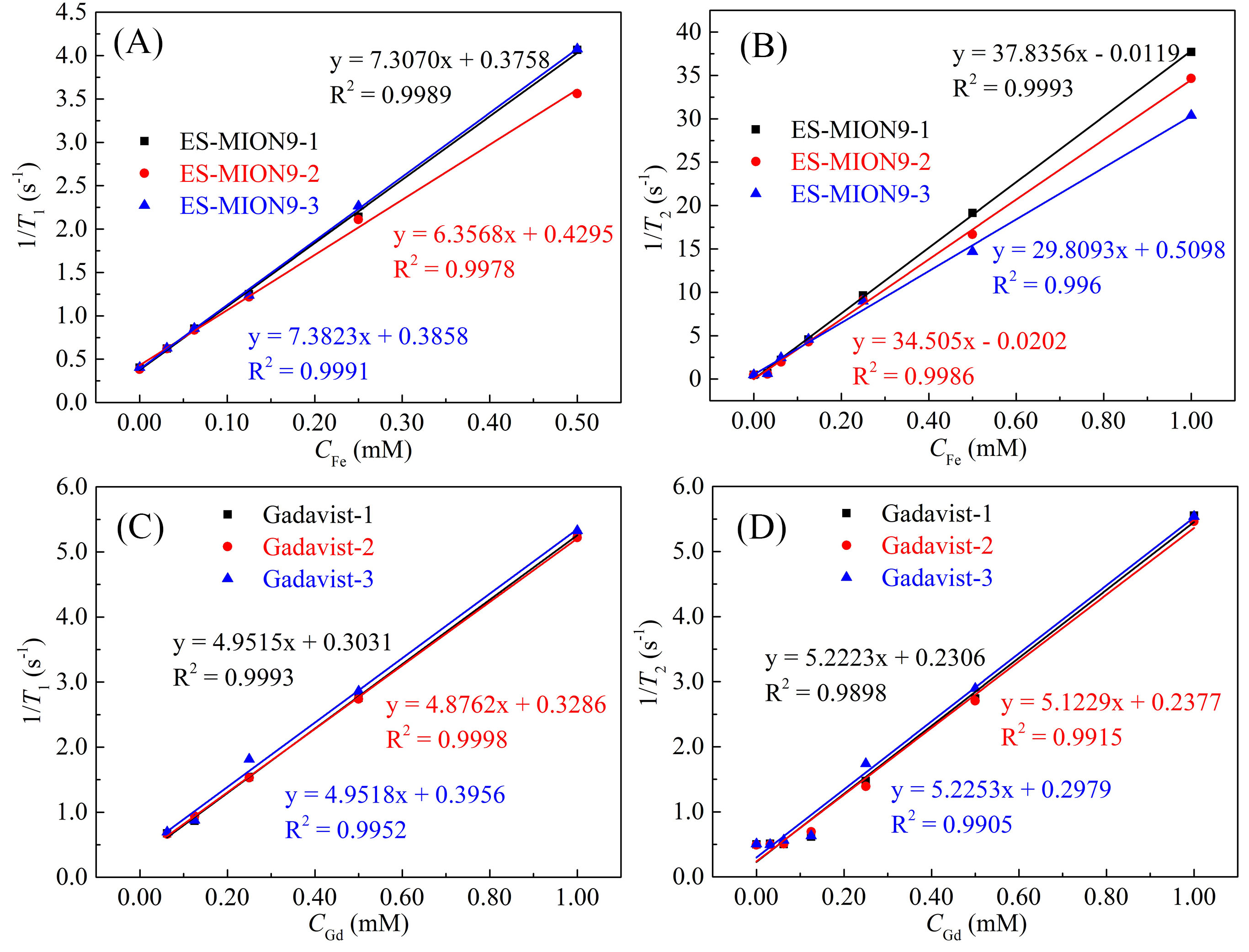


**Figure S2.** *T*_1_ relaxation rate (1/*T*_1_) (A, C) or *T*_2_ relaxation rate (1/*T*_2_) (B, D) plotted as a function of *C*_Fe_ or *C*_Gd_ for ES-MION9 (A, B) or Gadavist (C, D). Three different batches of ES-MION9 synthesized at same conditions and Gadavist solutions prepared from three different batches are measured to calculate the S.D. The magnetic field is 3.0 T.


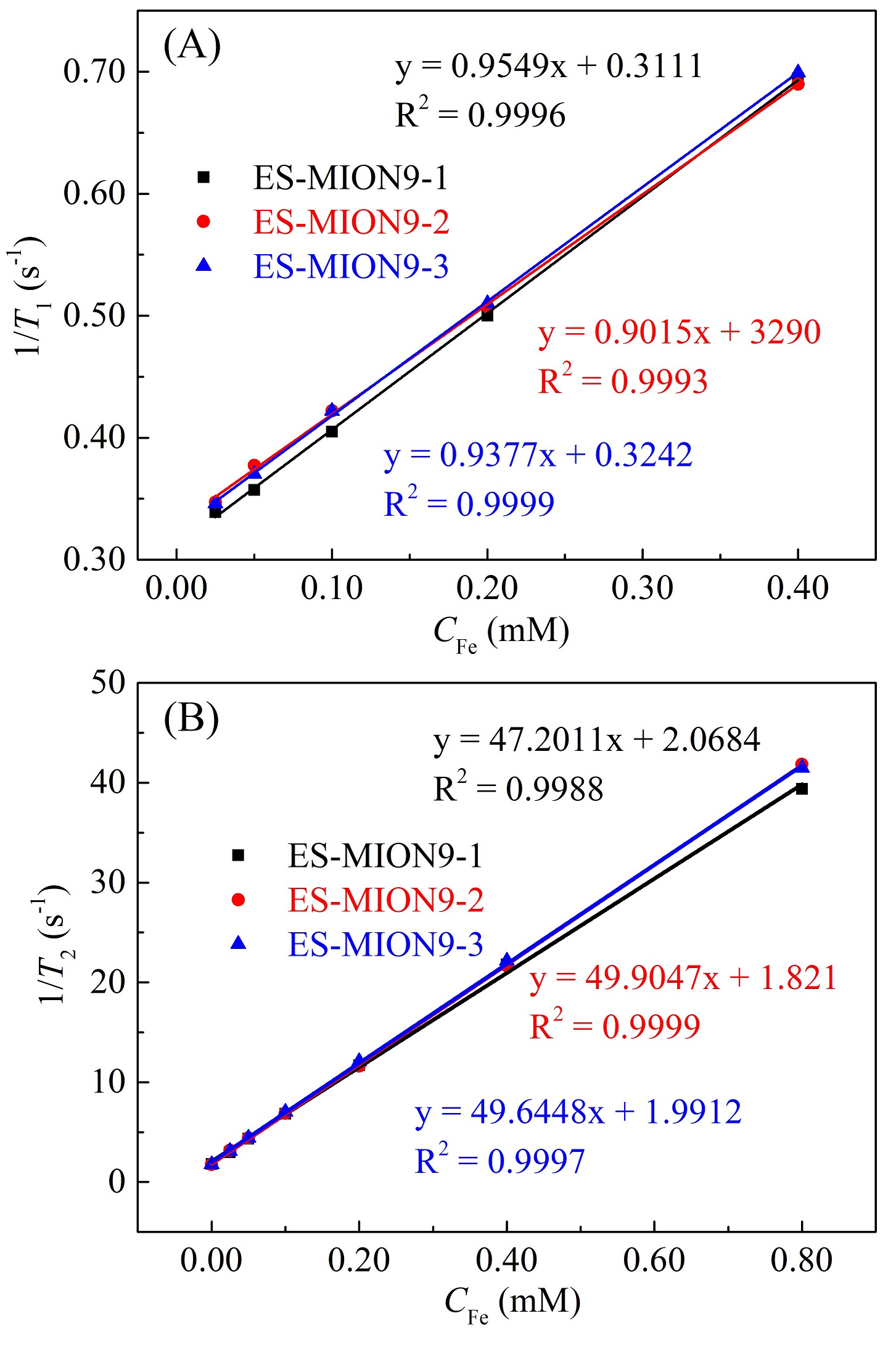


**Figure S3.** *T*_1_ relaxation rate (1/*T*_1_) (A) or *T*_2_ relaxation rate (1/*T*_2_) (B) plotted as a function of *C*_Fe_ for ES-MION9. Three different batches of ES-MION9 synthesized at same conditions are measured to calculate the S.D. The magnetic field is 7.0 T.


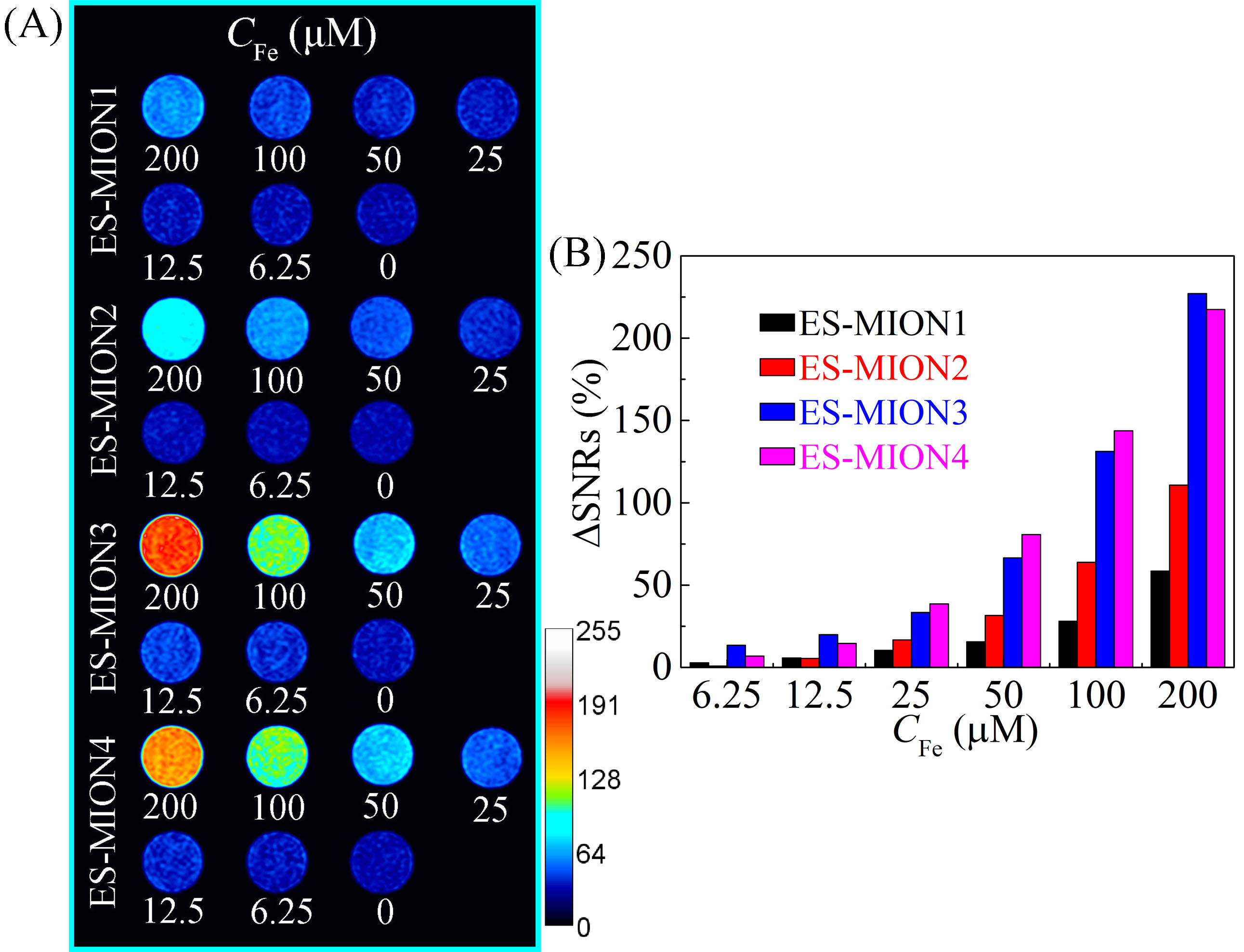


**Figure S4.** (A): *T*_1_-weighted MR images of ES-MION1-4 with various Fe (6.25 ~ 200 μM) (TE = 8.3 ms, TR = 200 ms). (B): ΔSNR of the MR images for ES-MION1-4 with various *C*_Fe_ as shown in (A), which was measured by the Image J. Magnetic field = 3.0 T.


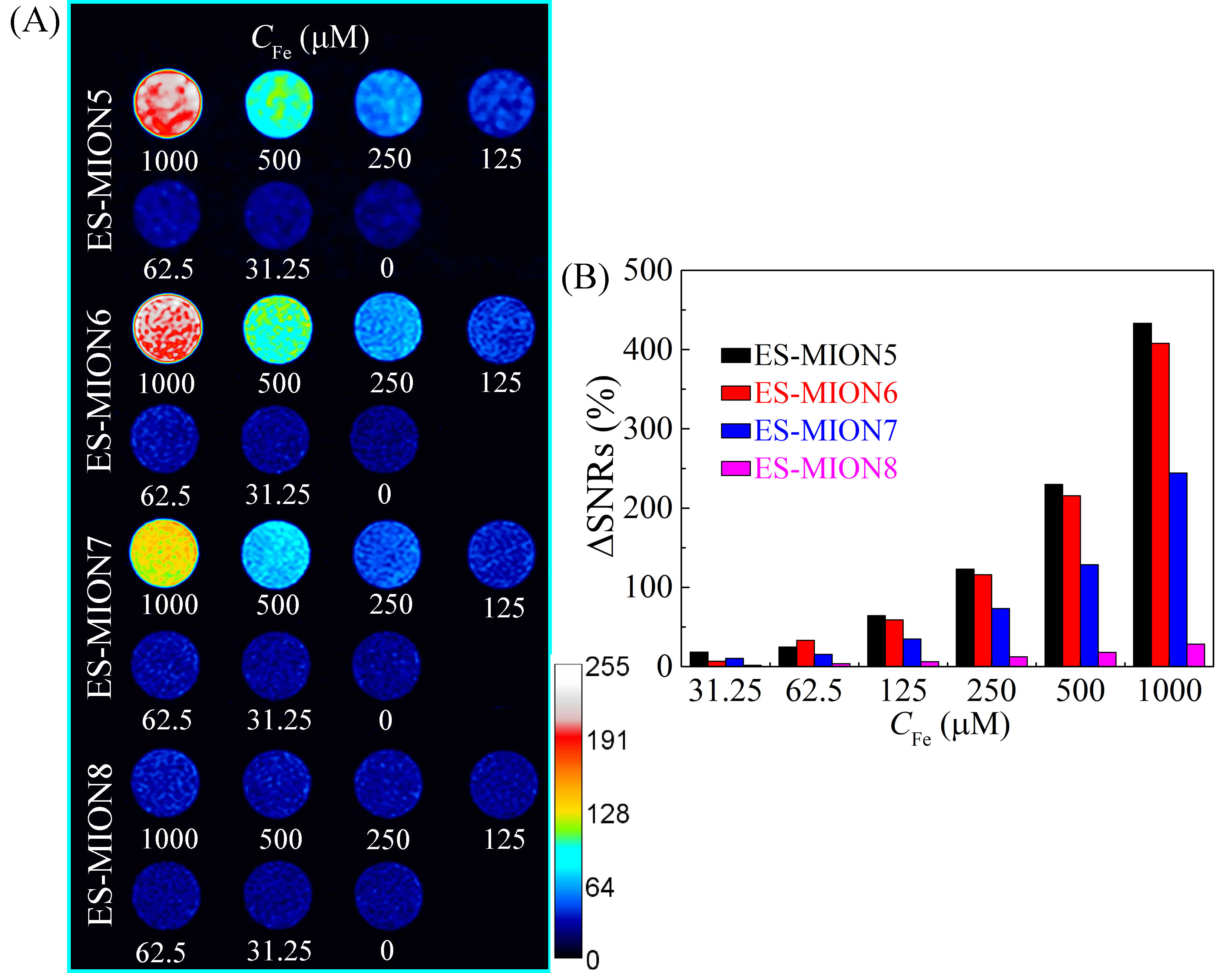


**Figure S5.** (A): *T*_1_-weighted MR images of ES-MION5-8 with various Fe (62.5 ~ 1000 μM) (TE = 8.3 ms, TR = 200 ms). (B): ΔSNR of the MR images for ES-MION5-8 with various *C*_Fe_ as shown in (A), which was measured by the Image J. Magnetic field = 3.0 T.


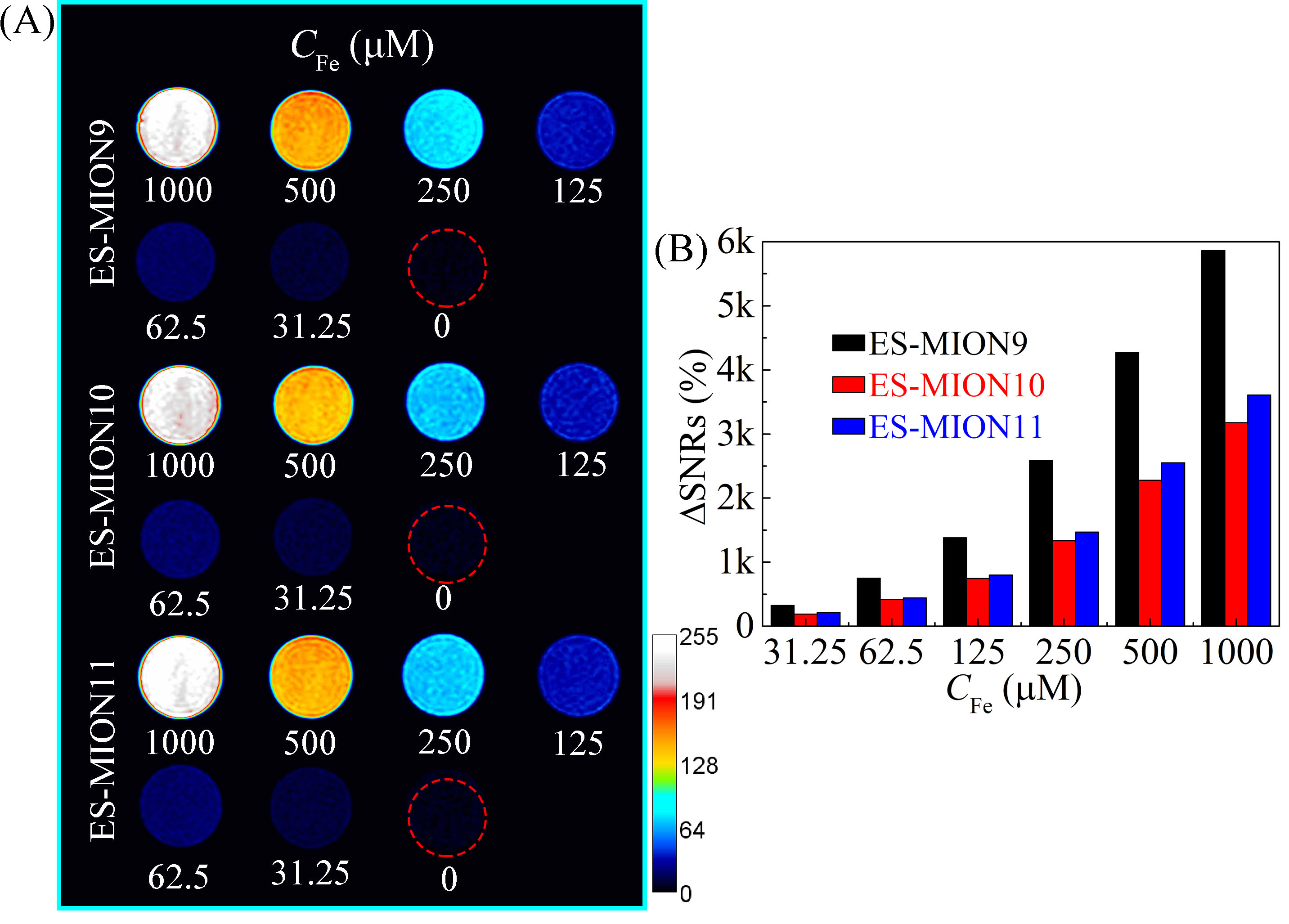


**Figure S6.** (A): *T*_1_-weighted MR images of ES-MION9-11 with various Fe (62.5 ~ 1000 μM) (TE = 8.3 ms, TR = 200 ms). (B): ΔSNR of the MR images for ES-MION9-11 with various *C*_Fe_ as shown in (A), which was measured by the Image J. Magnetic field = 3.0 T.


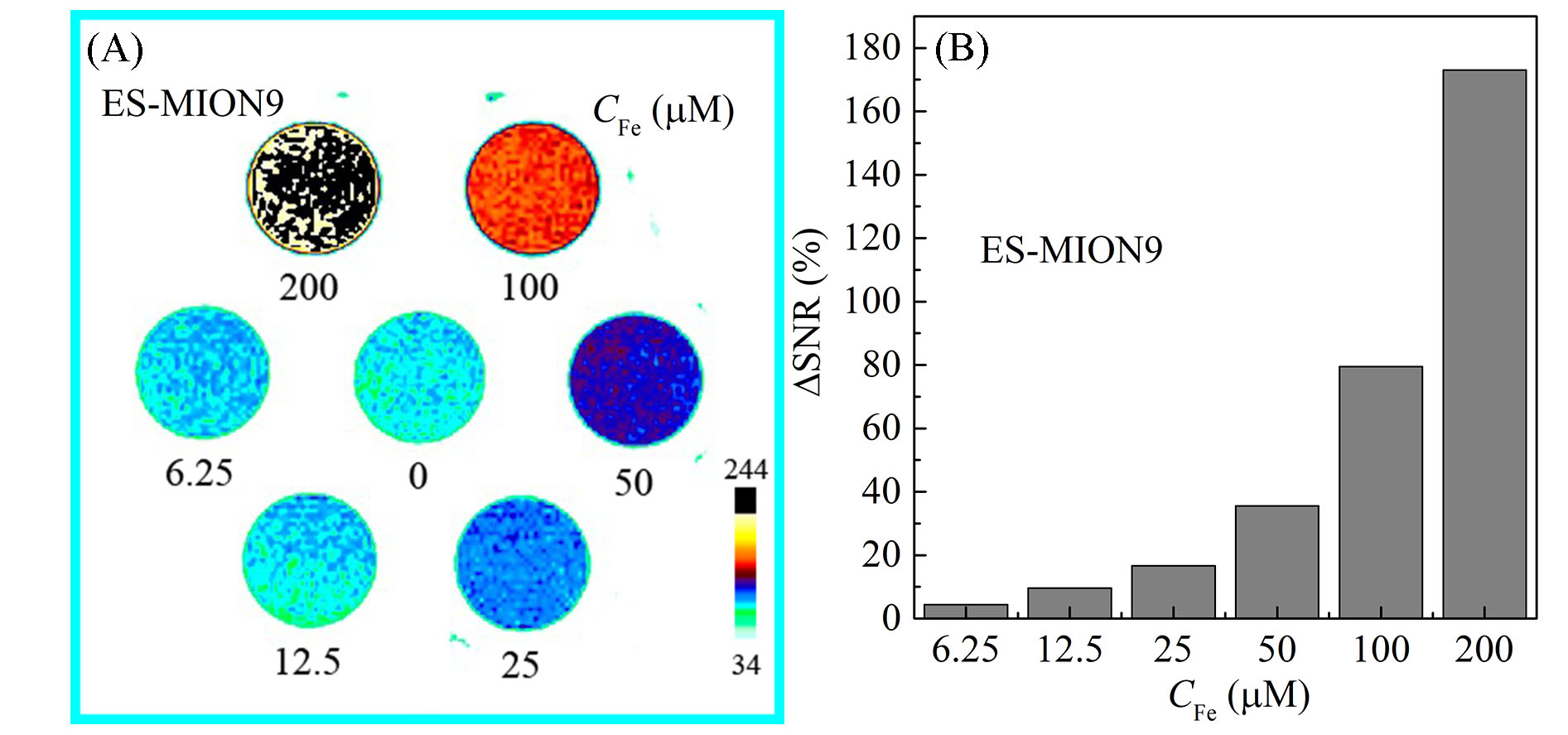


**Figure S7.** (A): *T*_1_-weighted MR images of ES-MION9 with various Fe (6.25 ~ 200 μM) (TE = 7.3 ms, TR = 200 ms). (B): ΔSNR of the MR images for ES-MION9 with various *C*_Fe_ as shown in (A), which was measured by the Image J. Magnetic field = 7.0 T.


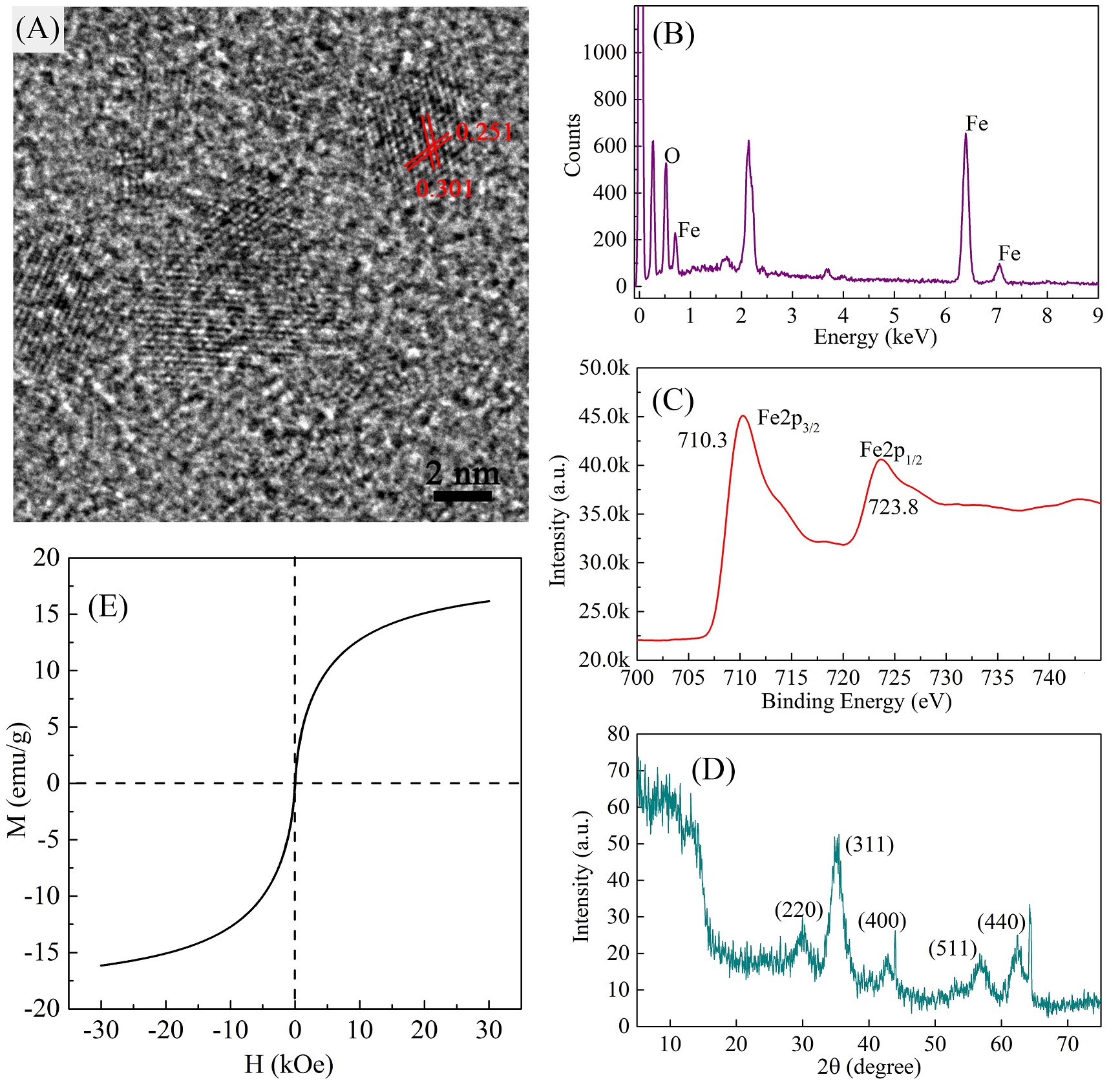


**Figure S8.** High resolution TEM (HR-TEM) image (A), energy dispersive X-ray spectrum (EDS) (B), X-ray photoelectron spectroscopy (XPS) (C), X-ray diffraction patterns (XRD) (D), and field-dependent magnetization curve (H-M) (E) of ES-MION9.


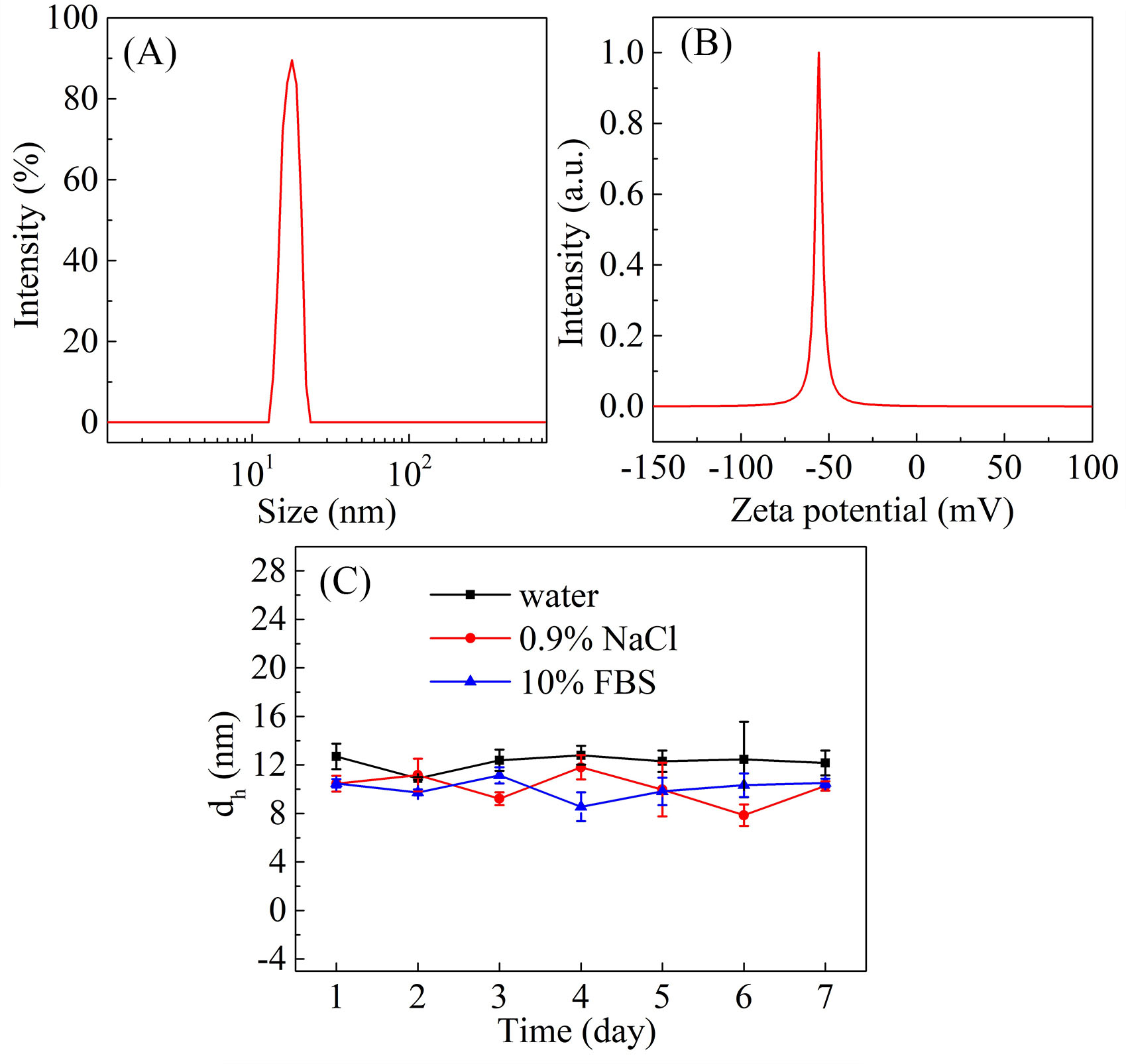


**Figure S9.** (A, B): Size distribution (A) or zeta potential (B) of ES-MION9 measured by DLS. (C): Hydrodynamic diameter (d_h_) changes of ES-MION9 (*C*_Fe_ = 1.0 mM) dispersed in pure water, 0.9% of NaCl, or 10.0% of fetal bovine serum (FBS) during storage at 4 ℃ (mean ± SD, n = 3).


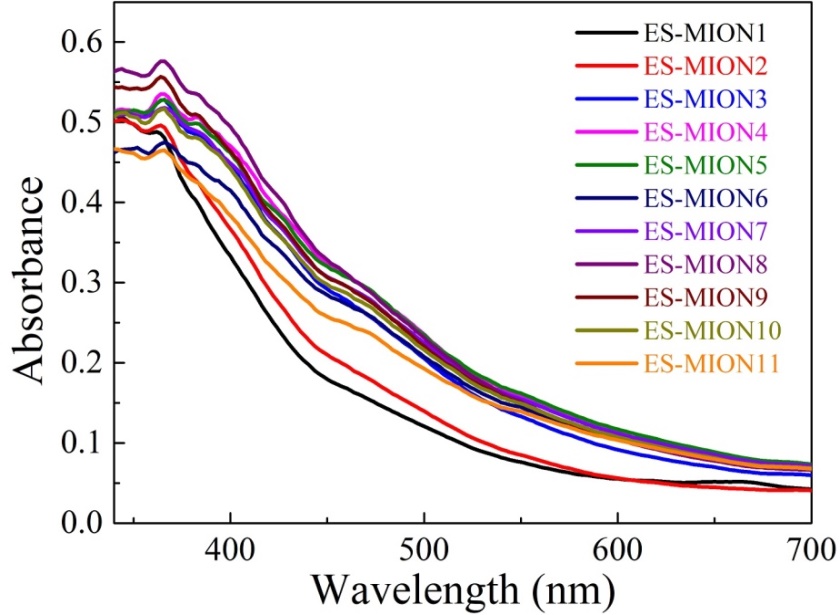


**Figure S10.** UV-vis spectra of ES-MION1-11 dispersions with a concentration of 1.0 mM Fe.


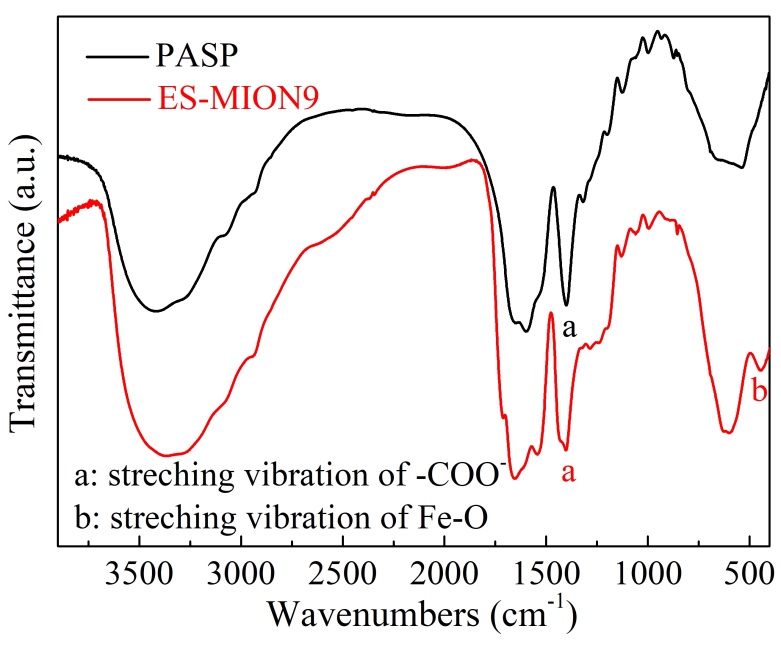


**Figure S11.** FT-IR spectra of PASP and the purified ES-MION9 stabilized with PASP.


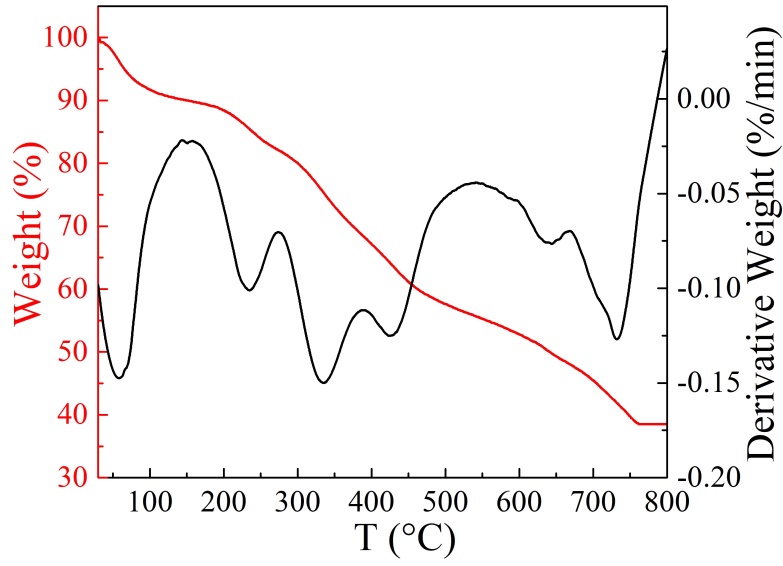


**Figure S12.** Thermogravimetry (TGA) and differential thermogravimetry (DTG) curves of ES-MION9.


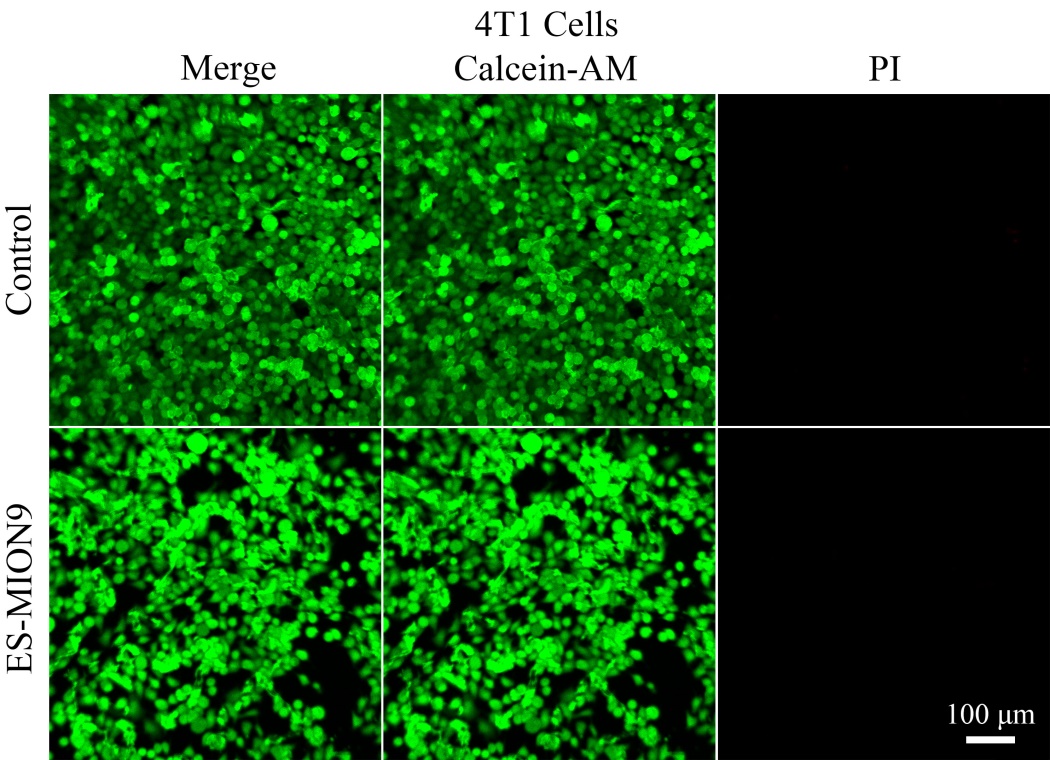


**Figure S13.** Live/dead staining of 4T1 cells after treatment with ES-MION9 for 24.0 h. Green represents the live cells, and red indicates the dead cells.


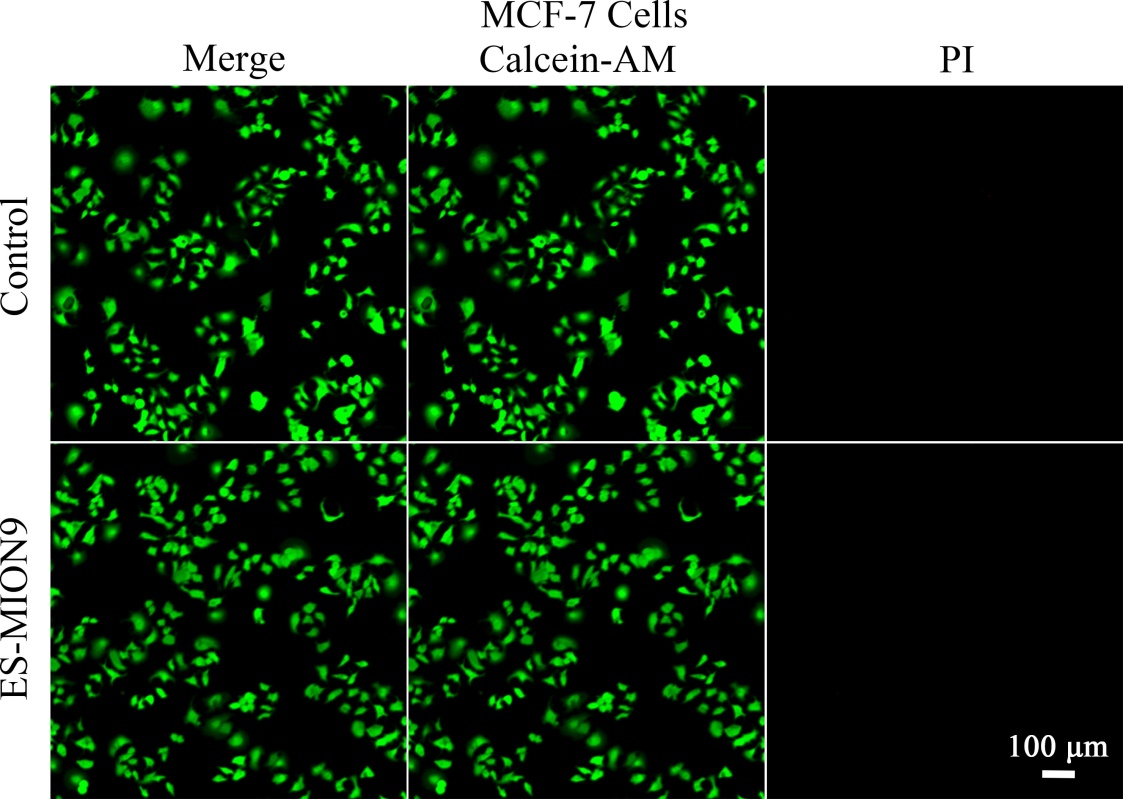


**Figure S14.** Live/dead staining of MCF-7 cells after treatment with ES-MION9 for 24.0 h. Green represents the live cells, and red indicates the dead cells.


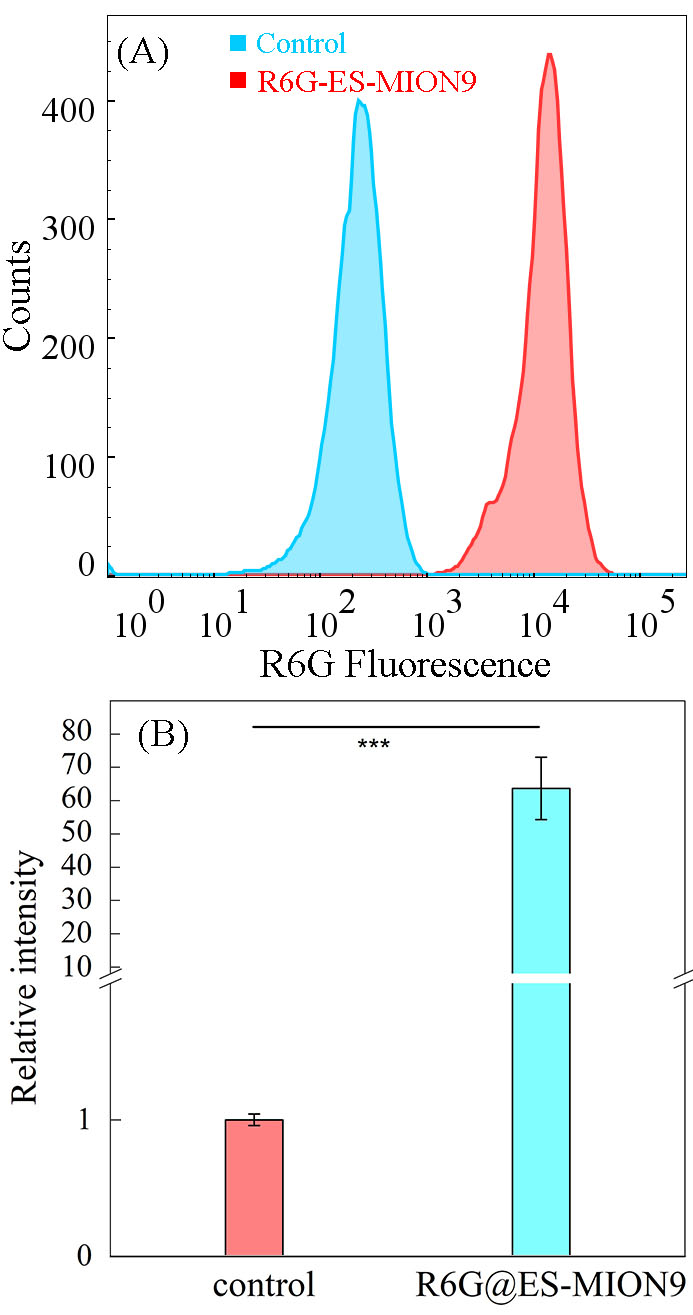


**Figure S15.** (A): Fluorescence distribution of 4T1 cells incubated with or without R6G@ES-MION9 analyzed by flow cytometry. (B): Relative intensity (*i.e.*, mean fluorescence intensity ratio) of R6G@ES-MION9-treated cells compared to untreated cells (control). Mean ± SD, *n* = 3. ***P < 0.001.


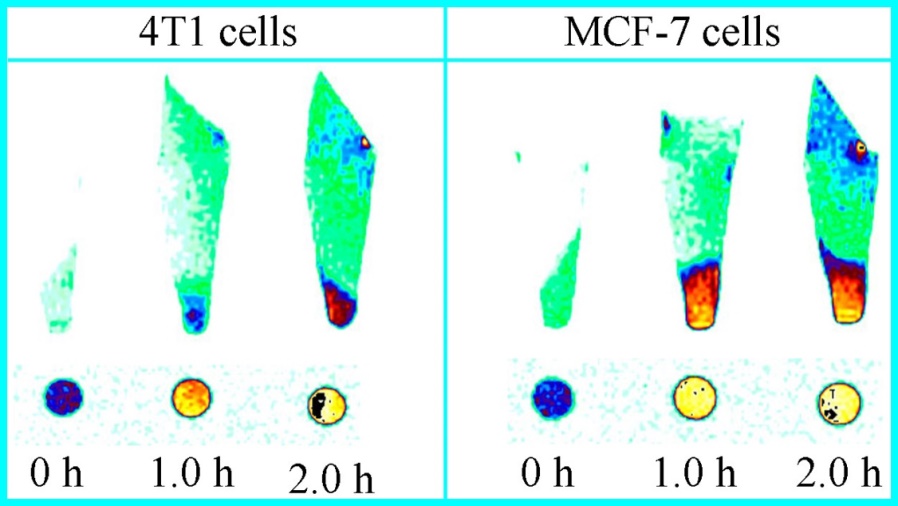


**Figure S16.** *T*_1_-weighted MR images of 4T1 cells or MCF-7 cells treated with ES-MION9 for 1.0 or 2.0 h. The cells untreated with ES-MION9 were used as controls. Magnetic field = 7.0 T.


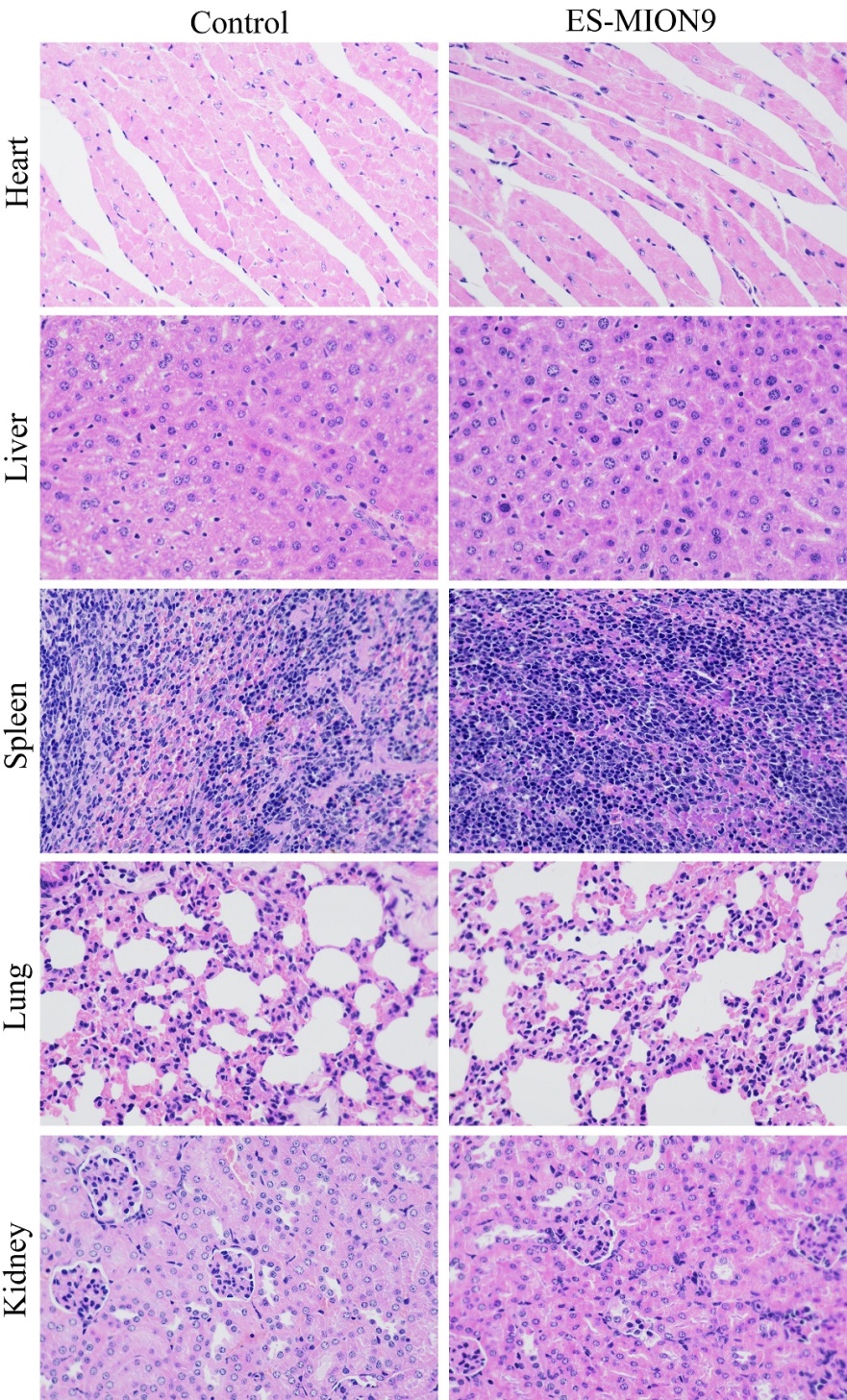


**Figure S17.** Representative optical microscopic pictures of the H&E-stained main organs from the normal mice without tumors (control), or that with intravenous injection of ES-MION9 (*C*_Fe_ = 5.0 mg/kg). The organs were collected at 3.0 days post injection. Comparing with the control, ES-MION9 did not exhibit obvious toxicity to the major organs, indicating good biocompatibility of our ES-MION9.
